# Toward reconstructing the evolution of advanced moths and butterflies (Lepidoptera: Ditrysia): an initial molecular study

**DOI:** 10.1186/1471-2148-9-280

**Published:** 2009-12-02

**Authors:** Jerome C Regier, Andreas Zwick, Michael P Cummings, Akito Y Kawahara, Soowon Cho, Susan Weller, Amanda Roe, Joaquin Baixeras, John W Brown, Cynthia Parr, Donald R Davis, Marc Epstein, Winifred Hallwachs, Axel Hausmann, Daniel H Janzen, Ian J Kitching, M Alma Solis, Shen-Horn Yen, Adam L Bazinet, Charles Mitter

**Affiliations:** 1 Center for Biosystems Research, University of Maryland Biotechnology Institute, College Park, Maryland 20742, USA; 2 Department of Entomology, University of Maryland, College Park, Maryland 20742, USA; 3 Laboratory of Molecular Evolution, Center for Bioinformatics and Computational Biology, University of Maryland, College Park, Maryland 20742, USA; 4 Department of Plant Medicine, Chungbuk National University, Cheongju 361-763, Korea; 5 Department of Entomology, University of Minnesota, St. Paul, Minnesota 55455, USA; 6 Department of Biological Sciences, University of Alberta, Edmonton, Alberta T6G 2E9, Canada; 7 Cavanilles Institute of Biodiversity and Evolutionary Biology, University of Valencia, Apartat de correus 2085, 46071 Valencia, Spain; 8 Systematic Entomology Laboratory, Agricultural Research Service, United States Department of Agriculture, Beltsville, Maryland 20705, USA; 9 Encyclopedia of Life, Smithsonian Institution, Washington, D.C. 20013-7012, USA; 10 Department of Entomology, Smithsonian Institution, Washington, D.C. 20013-7012, USA; 11 Plant Pest Diagnostics Branch, California Department of Food and Agriculture, 3294 Meadowview Road, Sacramento, California 95832-1448, USA; 12 Department of Biology, University of Pennsylvania, Philadelphia, Pennsylvania 19104, USA; 13 Bavarian State Collection of Zoology, Münchhausenstrasse 21, D-81247 München, Germany; 14 Department of Entomology, The Natural History Museum, Cromwell Road, London SW7 5BD, UK; 15 Department of Biological Sciences, National Sun Yat-Sen University, Kaohsiung 804, Taiwan

## Abstract

**Background:**

In the mega-diverse insect order Lepidoptera (butterflies and moths; 165,000 described species), deeper relationships are little understood within the clade Ditrysia, to which 98% of the species belong. To begin addressing this problem, we tested the ability of five protein-coding nuclear genes (6.7 kb total), and character subsets therein, to resolve relationships among 123 species representing 27 (of 33) superfamilies and 55 (of 100) families of Ditrysia under maximum likelihood analysis.

**Results:**

Our trees show broad concordance with previous morphological hypotheses of ditrysian phylogeny, although most relationships among superfamilies are weakly supported. There are also notable surprises, such as a consistently closer relationship of Pyraloidea than of butterflies to most Macrolepidoptera. Monophyly is significantly rejected by one or more character sets for the putative clades Macrolepidoptera as currently defined (*P *< 0.05) and Macrolepidoptera excluding Noctuoidea and Bombycoidea sensu lato (*P *≤ 0.005), and nearly so for the superfamily Drepanoidea as currently defined (*P *< 0.08). Superfamilies are typically recovered or nearly so, but usually without strong support. Relationships within superfamilies and families, however, are often robustly resolved. We provide some of the first strong molecular evidence on deeper splits within Pyraloidea, Tortricoidea, Geometroidea, Noctuoidea and others.

Separate analyses of mostly synonymous versus non-synonymous character sets revealed notable differences (though not strong conflict), including a marked influence of compositional heterogeneity on apparent signal in the third codon position (nt3). As available model partitioning methods cannot correct for this variation, we assessed overall phylogeny resolution through separate examination of trees from each character set. Exploration of "tree space" with GARLI, using grid computing, showed that hundreds of searches are typically needed to find the best-feasible phylogeny estimate for these data.

**Conclusion:**

Our results (a) corroborate the broad outlines of the current working phylogenetic hypothesis for Ditrysia, (b) demonstrate that some prominent features of that hypothesis, including the position of the butterflies, need revision, and (c) resolve the majority of family and subfamily relationships within superfamilies as thus far sampled. Much further gene and taxon sampling will be needed, however, to strongly resolve individual deeper nodes.

## Background

The Lepidoptera (butterflies and moths) are one of the four mega-diverse insect orders, containing over 165,000 described species [1,2]. Primarily plant-feeding as larvae and nectar-feeding as adults, they are a prominent element of terrestrial ecosystems, functioning as herbivores, pollinators and prey, as well as constituting one of the most damaging groups of pests overall to agriculture. Lepidoptera have served as important model systems for studies of genetics, physiology, development, and many aspects of ecology and evolutionary biology including insect/plant coevolution [3]. As conspicuous terrestrial invertebrates, they have become central as well to ecosystem assessment, conservation planning, and public outreach designed to foster environmental awareness [4].

A phylogenetic framework is fundamental to all attempts at understanding the diversity, adaptations and ecological roles of Lepidoptera. Deep-level lepidopteran phylogeny, however, remains largely a mystery, except in the species-poor, basal ("non-ditrysian") lineages (review in [3]). Monophyly seems well established for many of the 47 superfamilies but not for all, and phylogeny within superfamilies has only begun to receive concerted study. Relationships among superfamilies have rarely been examined. In the clade Ditrysia, which contains over 98% of lepidopteran species and 80% of the families, the most authoritative phylogenetic hypothesis to date postulates only 11 tentative monophyletic groupings among the 33 superfamilies [1], and is not based on a quantitative phylogenetic analysis.

In this paper we present an initial study undertaken to help guide the design of a very large molecular investigation of lepidopteran phylogeny now in progress (700+ exemplars, 5-26 genes; see http://www.Leptree.net/). We test the ability of five protein-coding nuclear genes (6.7 kb total) to resolve relationships among 123 species, drawn from 27 superfamilies and 55 families that together contain nearly 90% of the species of Ditrysia. We then compare the results to previously postulated relationships, most of which are based on morphology. This report presents by far the largest explicit character-based analysis of ditrysian phylogeny yet published, though others are underway (L. Kaila, personal communication; see http://www.leptree.net/community_directory).

The working hypothesis that our sampling is designed to test is the compendium of expert opinion on within- and among- superfamily relationships compiled by Kristensen [5]. The major divisions follow Minet [6], who recognized three successively more restricted clades within Ditrysia. In order from most to least inclusive, these are Apoditrysia, Obtectomera, and Macrolepidoptera. These divisions, based on morphological characters, are correlated with broad postulated trends in life history [7,8]. For example, in most non-ditrysian and many primitive ditrysian lineages, the larvae typically live and feed inside the host plant, most often as leaf miners, emerging, if at all, only to pupate. In the majority of non-macrolepidopteran Apoditrysia, the larvae live outside the plant but construct and feed within shelters such as silk webs or leaves rolled and tied with silk. Only in the putative clade Macrolepidoptera, comprising the butterflies and larger moths and totaling about 100,000 described species in 33 families and 11 superfamilies, do the larvae - "caterpillars" - typically feed exposed on the leaf. Macrolepidoptera also differ from the remaining Ditrysia, often referred to informally as "Microlepidoptera," in that over 80% of the species, mainly in the superfamilies Drepanoidea, Geometroidea, and Noctuoidea, possess some form of ultrasound-detecting "ears," most often located on the thorax or abdomen. These hearing organs probably evolved as a defense against bats that hunt using sonar [9]. Among the microlepidopterans, in contrast, the only large superfamily to possess such "ears" is Pyraloidea, though similar structures have been described in two other small groups [10,11]. Rigorously documenting (or disproving) the existence of such major transitions and their evolutionary consequences is a central long-term goal of our studies of lepidopteran phylogeny.

The early radiations of ditrysian superfamilies are likely to have been rapid [2], and to have taken place mainly during the Cretaceous [12], a problematic time span for insect molecular phylogenetics (e.g., [13]). Cretaceous divergences are sufficiently young that most coding sequences, even ones chosen specifically for suitability over this time span, have accumulated relatively few amino acid changes. Thus, many genes will probably be needed to provide sufficient non-synonymous signal to resolve a rapid radiation. At the same time, these divergences are sufficiently old that sites undergoing synonymous substitutions, the largest source of signal, will be both approaching saturation and diverging in base composition, posing special difficulties for phylogeny inference. Resolution of ditrysian relationships is thus likely to require, in addition to very large character sets, an especially careful choice among analytical methods, to extract the maximum amount of signal from a highly heterogeneous and challenging set of relevant characters. For this reason, our study included extensive exploration of the differing properties and phylogenetic signal content of different character subsets. We focus particularly on the distinction between sites undergoing non-synonymous versus synonymous substitution, and on the computational effort required to extract full information from these.

## Methods

### Taxon sampling and specimen acquisition

Complete coverage of the lepidopteran families and superfamilies, many of which contain just a few, difficult-to-collect species, is beyond the reach of this initial study. Our more modest aim here was simply to represent a majority of the probable major lineages of Ditrysia. The distribution of our exemplars across the major clades of Minet [6] is shown in Additional file 1, which also lists families/superfamilies not sampled, to illustrate the extent of our coverage. Our sampling, which builds on a preliminary study of macrolepidopteran (especially bombycoid) relationships [14], is most dense in Macrolepidoptera (66 exemplars; 11 of 11 superfamilies) and non-macrolepidopteran Obtectomera (17 exemplars; 4 of 6 superfamilies), which together contain about 70% of ditrysian species diversity. Thirty species of non-obtectomeran Apoditrysia are included, representing eight of eleven superfamilies, and seven species of non-apoditrysian Ditrysia representing four of five superfamilies. One of the latter, Tineoidea (two species included), was used to root the tree, as tineoids are generally agreed to be the oldest superfamily of Ditrysia [1,6,15]. We sampled relatively extensively within a few larger superfamilies, both to get an adequate estimate of ancestral character states, and to further test the resolving power of our genes within superfamilies; our main focus, however, is among-superfamily relationships. Altogether our sample includes 27 of 33 superfamilies and 55 of 100 families of Ditrysia. The six superfamilies not represented each contain fewer than 100 species. The missing families are likewise mostly species poor, the main exceptions being Lycaenidae and several large families of Gelechioidea. Thus, the families represented in our study contain the great majority (>85%) of all species of Ditrysia. The classification system used (Additional file 1) follows the authorities in Kristensen [5], with exceptions as noted, including the following: in Pyraloidea we follow the more recent classification of Solis and Maes [16]; in Geometridae we update the classification following Hausmann [17], Holloway [18], Scoble [19] and Young [20].

Specimens for this study, obtained with the kind help of collectors around the world (see Acknowledgements) are stored in 100% ethanol at -85°C as part of the ATOLep collection at the University of Maryland (details at http://www.leptree.net/collection). DNA extraction used only the head and thorax for most species, leaving the rest of the body including the genitalia as a voucher (see Additional file 1). Wing voucher images for all adult exemplars are posted at http://www.leptree.net/voucher_image_list, and DNA 'barcodes' for nearly all specimens have been kindly generated by the All-Leps Barcode of Life project http://www.lepbarcoding.org/, allowing check of our identifications against the BOLD (Barcode of Life Data system) [21] reference library and facilitating future identification of specimens whose identity is still pending (i.e., species listed as 'sp.' or 'unidentified' in this report).

### Gene sampling and generation of DNA sequence data matrices

Our sequence data come from five protein-coding nuclear gene regions, identical to those used by Regier et al. [14], with demonstrated promise for resolving deeper lepidopteran relationships. The combined length of these regions after alignment is 6867 nucleotides (nt) per taxon, or 6759 if 108 characters (1.6% of total) around indel regions are excluded because of uncertain alignment. The five sequences are: *CAD *(2928 nt; contributes 43% of total sequence in this study) [22], *DDC *(1308 nt; 19% of total) [23], *enolase *(1134 nt; 17% of total) [24], *period *(987 nt; 15% of total) [25], and *wingless *[26] (402 nt; 6% of total).

Why choose these genes and exclude markers more commonly used in Lepidoptera? We used protein-coding regions rather than ribosomal RNA sequences because they are easier to align and their sequence evolution is easier to model. We used nuclear rather than mitochondrial protein-coding genes because the faster evolution of the latter, and their extreme base composition bias, render them less suitable for recovering deeper divergences [27]. For the same reasons, we did not include the "barcode" fragment of mitochondrial *CO-I *in our analyses. We did not sequence *EF-1α*, the nuclear protein-coding gene most often studied in Lepidoptera, because in comparisons between superfamilies, synonymous substitutions in this gene approach saturation, while amino acid changes are too few to provide much information [28]. We used RT (Reverse Transcription)-PCR, instead of the more standard genomic DNA PCR, to avoid amplification of introns. This increases the efficiency of obtaining coding sequences, which are easier to interpret than introns and likely to carry on average more information on deeper divergences than introns. We intend no criticism of previous uses (including our own) of genes and gene regions excluded here, which have yielded countless valuable results at lower taxonomic levels. Rather, our present choice of markers was motivated simply by the goal of obtaining as much information about deeper relationships as possible from the finite resources available for this project.

A detailed protocol of all our laboratory procedures has been published ([30]; see online Appendix 2 therein). Further descriptions, including gene amplification strategies, PCR primer sequences, and sequence assembly and alignment methods, can be found in previous papers ([14,29,30]; see online supplementary materials therein). To summarize, total nucleic acids were isolated and specific regions of the cognate mRNAs were amplified by RT-PCR. Specific bands resulting from RT-PCR were gel-isolated and reamplified by PCR using hemi-nested primers, except for wingless, which lacks nested primers. Visible bands that were too faint to sequence were reamplified using the M13 sequences at the 5' ends of all primers. PCR amplicons were sequenced directly on a 3730 DNA Analyzer (Applied Biosystems). Sequences were examined, edited and assembled using the TREV, PREGAP4, and GAP4 programs in the STADEN package [31]. Multiple sequence alignments were made manually in Genetic Data Environment [32], and a data-exclusion mask of 108 nucleotides was applied. Alignment was generally straightforward, given the overall conservation of the protein sequences. The entire aligned data matrix in Nexus format is available in Additional file 2: "Data matrix". GenBank numbers as well as genes and taxa for which sequences are partial or missing are listed in Additional file 1.

### Congruence among genes

Most of our analyses were based on the maximum likelihood criterion applied to nucleotides, as implemented in GARLI (Genetic Algorithm for Rapid Likelihood Inference; version 0.951) [33]. We selected the best-fitting substitution model (invariably GTR+ G + I) under the Akaike information criterion (AIC), using Modeltest version 3.7 [34]. To determine whether conflicting signal among genes might significantly complicate phylogenetic analysis, we first conducted a bootstrap analysis (1000 pseudo-replicates) for each gene separately (all nucleotides), looking for groupings that conflicted moderately or strongly (bootstrap support of 70% or more) with other individual genes, with the all-gene result, or with conventional understanding of relationships. The results are presented and discussed in Additional file 3. Only two such conflicts were found, one previously reported [14], both of which involved within-family relationships. Given the rarity of inter-gene conflict, we felt justified in concatenating all five genes into a single data set for estimating the organismal phylogeny.

### Character sets differing in synonymous versus non-synonymous change

While heterogeneity among genes does not appear to present a major obstacle to analysis of these data, variation among sites within genes is potentially a larger problem. Most phylogeny reconstruction methods for nucleotides, including those used here, assume that the relative rates of different nucleotide substitution types (i.e., the instantaneous rate matrix) are constant across the tree, and can be led astray if this assumption is violated. In our experience [14,29], strong compositional heterogeneity, an indicator of underlying process heterogeneity, is common at sites capable of undergoing synonymous substitution. For this reason, we explored several character sets which differ strongly in the likely proportion of synonymous change. In a subsequent section (see below) we compared compositional heterogeneity among character sets and its possible consequences.

To create a character set essentially free of synonymous change, we first isolated from the total population of first codon position sites (nt1) a subset which we term "noLRall2," containing all sites belonging to codons in which no more than one leucine or arginine occurred among the taxa considered. Since only leucine (L) and arginine (R) codons can undergo synonymous change through a single nucleotide substitution at nt1, variation in the "noLRall2" subset should reflect mostly non-synonymous change. Combining noLRall2 with second codon position sites (nt2) produces a character set we term "noLRall2 + nt2," which should contain most of the total signal from non-synonymous change. The "noLRall2" subset of nt1 contributes 150 more sites to the non-synonymous category than does the similarly-motivated "noLR1" subset of Regier et al. [14], which excluded all sites at which even one leucine or arginine occurs. The nt1 subsets were generated using a Perl script available in Regier et al. ([29]; see online Appendix 4 therein). As a more conventional estimate of predominantly non-synonymous signal we also analyzed nt1 + nt2 alone. As an estimate of almost entirely synonymous signal we first analyzed nt3 alone. A second, larger set of mostly synonymous change was then obtained by combining nt3 with a character set we call "LRall2", which is the complement to "noLRall2" above. That is, "LRall2" consists of nt1 sites at which either leucine or arginine (or both) occur in two or more taxa, opening the potential for those taxa to differ by synonymous substitutions. Analysis of all nucleotides together (nt123) provides a third data set dominated (>90%) by synonymous change.

### Tree space and the efficacy of ML searches

For reasons developed in the Discussion, we spent considerable effort on obtaining the best possible tree estimate, as well as gauging bootstrap support for that estimate. We first sought to gain some idea of how many heuristic searches might be required to consistently obtain an optimal or near-optimal tree. To this end, we first ran 10,000 GARLI searches on each character set, using the default search parameters. We regarded the best-score resulting tree(s) from this very large effort to represent the best topology that it is feasible to obtain with our computing resources. While we have no way of knowing whether this is actually the globally best tree, the chance that it is so increases with the number of times the same topology is recovered in 10,000 trials.

To determine how many replicates to include in subsequent searches, we used the number of times (N) the "best feasible" topology appeared in the 10,000 replicate searches to estimate how many search replicates would be needed to be 95% confident of getting the "best" topology at least once. In other words, for what number of replicates would the chance of never getting the "best" topology be only 5%? From the formula probabilities under the binomial distribution, that number can be expressed as x in the equation (1-N/10,000)^x ^= 0.05, where N is the number of trials, among the 10,000 trials, that yielded the best-score topology. Estimates of the number of runs needed ranged from about 40 to nearly 15,000, depending on the character set. For all subsequent analyses of each data set (see below), we used the number of GARLI runs thus specified for estimating the optimal topology. Node support for each character set was gauged by performing 1000 bootstrap replicates under default search parameters. For consistency in the characterization of results, we will refer to bootstrap support of 70-79% as "moderate" and support ≥ 80% as "strong."

We also used the results of the 10,000 heuristic searches for each data set to explore the nature of tree space in the vicinity of the best feasible topology. To gauge the tightness of clustering around the best tree, we computed the topological difference from the best topology for each of the 10,000 trees, defined as the number of nodes collapsed in its strict consensus with the best tree. This is a variant of the consensus fork index of Colless [35]. We also examined the frequency distribution among the 10,000 trees of departure in likelihood score from the best score, expressed as a percent of the best score, and looked for a relationship between topological difference and likelihood score difference.

The total number of GARLI runs for the entire study amounted to about 100,000. To make this large effort possible we used grid computing [36] through The Lattice Project [37], which includes clusters and desktops in one encompassing system [38]. A grid service for GARLI was developed using a special programming library and associated tools [39]. Following the model of Cummings et al. [40,41], we distributed required files among hundreds of computers, where the analyses were then conducted asynchronously in parallel.

### Bayesian analysis

Although analyzing character sets separately can help elucidate the evolutionary properties of each, for phylogeny inference one would ideally analyze complementary character sets simultaneously, taking into account the different substitution behavior of each. GARLI does not yet allow data partitioning under the GTR + G + I model. For this reason, we also performed a Bayesian analysis, with the full data set (nt123) partitioned into site populations undergoing largely non-synonymous (noLRall2 + nt2) versus largely synonymous (LRall2 + nt3) change. This analysis used MrBayes (version 3.2; parallel processing enabled) [42,43]. Default values were used for the prior probability distribution of the parameters of the likelihood model (GTR + I + G), except that the rate multipliers of the character sets were specified to be variable. For all character sets the gamma shape parameter, proportion of invariable sites, character state frequencies, and substitution rates of the GTR + G + I model were unlinked. The Markov chain Monte Carlo analysis was run for 111 million generations, and samples were taken every 1000 generations. The analysis consisted of two simultaneous, independent runs with four chains, and samples from the two runs were pooled for the final result. All samples were taken after the two runs had converged, as indicated by a standard deviation of split frequencies smaller than 0.01 and diagnostic analyses implemented in AWTY [44]. Convergence was first seen in the 92 millionth generation.

### Testing significance of non-monophyly of predicted groupings

Several groupings proposed in the composite working hypothesis [5] were non-monophyletic in all of our analyses. We used the Approximately Unbiased (AU) test of Shimodaira [45] to determine whether our data or subsets thereof significantly reject those previous hypotheses, against the alternative that the discrepancy can be explained by sampling error in the sequence data. The test determines whether the best tree possible under the constraint of monophyly, no matter what its topology may be otherwise, is a significantly worse fit to the data than the best tree without that constraint. Table 1 lists the eight groups tested for significance of non-monophyly. For each combination of one character set and one group of uncertain monophyly, we performed a GARLI analysis under the constraint of monophyly for the group in question, and a parallel unconstrained analysis. Each analysis used the number of GARLI runs determined to be appropriate for that character set as described above. The site likelihoods of the resulting best constrained tree and best unconstrained tree were then estimated with PAUP* [46], and the trees and site likelihoods for all comparisons combined into a single input file for the CONSEL package [47]. In CONSEL, the test statistic of Shimodaira [45] was used to determine the difference in fit to data of the constrained and unconstrained trees, and the bootstrapping procedure of Shimodaira [45] was used to determine the significance of those differences.

**Table 1:** Tests for significance of non-monophyly (AU test of Shimodaira [45]) for some predicted clades not recovered in the ML trees.

Predicted clade	*P *values		
	
	noLRall2 + nt2 (31 heuristic searches)	nt12 (264 heuristic searches)	nt123 (224 heuristic searches)
Apoditrysia	0.121	0.312	0.119

Obtectomera	0.192	0.364	0.700

Macrolepidoptera	0.106	0.020	0.183

Butterflies *sensu *Scoble + Geometroidea + Drepanoidea + Cimelioidea + Calliduloidea	0.004	0.001	0.004

Bombycoidea + Lasiocampoidea + Mimallonoidea	0.473	0.460	0.418

Drepanoidea	0.236	0.077	0.240

Noctuoidea	0.377	0.172	0.342

Papilionoidea	0.521	0.389	0.382

### Base composition heterogeneity and its consequences

One of the most difficult problems that can affect molecular phylogenetic inference is among-lineage heterogeneity in nucleotide base composition [48]. To investigate the potential influence of compositional heterogeneity in our character sets, we first conducted chi-square tests of among-taxon heterogeneity on a character set undergoing mostly non-synonymous change, noLRall2 + nt2, and on a character set undergoing mostly synonymous change, nt3. In each case, the test was performed both on the entire character set, and after elimination of invariable sites in that character set. To gauge the level of taxonomic divergence over which compositional heterogeneity becomes apparent, we carried out these tests both for all taxa together and for 13 taxon subsets (families, superfamilies or related sets thereof). To explore the possible consequences for phylogeny inference of the compositional heterogeneity revealed by these tests, we compared neighbor joining trees computed from three distances that are influenced to different degrees by compositional heterogeneity: (a) LogDet distances, which are relatively insensitive to compositional heterogeneity [49]; (b) ML distances based on the GTR model, which can be influenced by compositional heterogeneity; and (c), Euclidean distances calculated on the proportions of the four nucleotide states treated as independent characters, which will reflect only compositional heterogeneity. These calculations were performed separately on noLRall2 + nt2, nt123, and nt3. The calculations were carried out with PAUP* [46], except that the Euclidean distances were generated using MBE Toolbox V2.2 [50], after modification of the source code to correct for varying sequence lengths.

## Results

### Tree space and the efficacy of ML searches

The best-score topology occurring one or more times in a set of 10,000 GARLI search replicates, taken here to represent the closest feasible estimate to the globally best tree, was found with a frequency (in those 10,000 searches) that differed markedly among data sets and appeared inversely correlated with the prevalence of synonymous substitutions. The "best-feasible" topology was found 925 times for the largely non-synonymous-change character set noLRall2 + nt2, 133 for nt123, 114 times for nt12, and only twice in 10,000 trials for nt3; change in the latter is almost entirely synonymous. From these numbers, we estimate (see Methods) that to be 95% confident of getting the "best-feasible" topology at least once in a new analysis, one would need to perform 31 GARLI search replicates for noLRall2 + nt2, 224 for nt123, 264 for nt12, and 14,978 for nt3 alone. The result for nt3 strongly suggests that our searches did not find the globally optimal tree(s).

**Figure 1:**
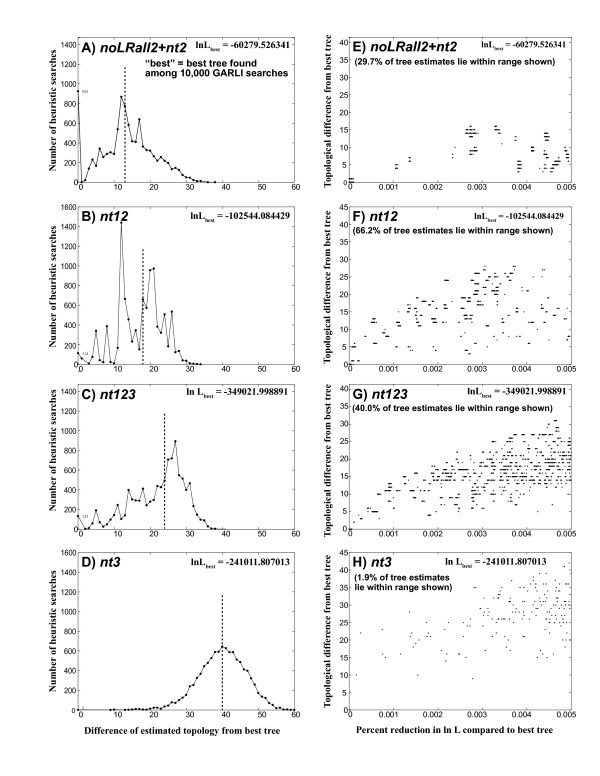
**Characterization of tree populations resulting from very-extensive heuristic ML searches (10,000 GARLI runs) on four character sets**. For each character set there are two side-by-side graphs: A, E for noLRall2 + nt2; B, F for nt12; C, G for nt123; D, H for nt3. The left-hand graph plots the number of searches resulting in a tree with given topological difference score (Y axis), against the topological difference itself (X axis), where "topological difference" for each search result tree defined as the number of nodes collapsed in the strict consensus between that tree and the overall best tree ("best" tree). A dashed vertical line marks the median topological difference for each data set. The right-hand graph plots topological difference from the overall best tree (Y axis) against difference in ln L from the overall best tree (X axis), expressed as a percent of the best score, for all trees with likelihood scores within 0.005% of the best.

We also used the results of the 10,000 heuristic searches for each data set to explore the nature of "tree space" in the vicinity of the best tree (Fig. 1). The 10,000 GARLI trees showed a broad range of topologies, and the differences of these topologies from the best tree appear correlated, across data sets, with the fraction of synonymous change (Fig. 1, left column). For example, the topology with the median degree of difference from the best topology (dotted lines in Fig. 1) differs from the best topology in 14 of 120 nodes for noLRall2 + nt2, 18 nodes for nt12, 24 nodes for nt123, and 40 nodes for nt3. In general, very large numbers of trees differ only slightly in likelihood score from the best tree, yet these trees can differ dramatically in topology (Fig. 1, right column). For example, 40% of all 10,000 trees for nt123 have likelihood scores within 0.005% of the maximum score, yet these can differ from the best tree at up to 30 nodes. Tree score difference is only loosely correlated with topological difference. For example, trees for nt12 that differ in score from the best by between 0.003% and 0.004% span a broad range of topological differences from the best tree, from five to 27 nodes not shared (Fig. 1F).

### Phylogeny estimates from the four character character sets

**Figure 2:**
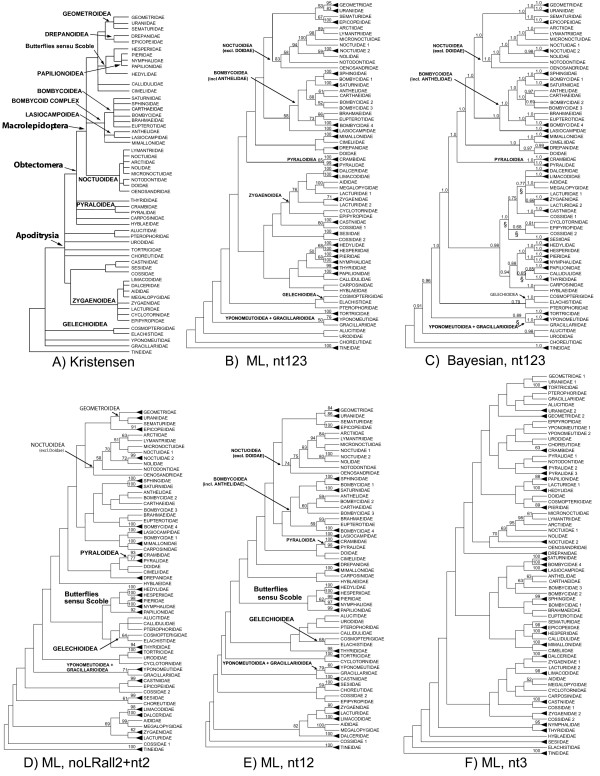
**Relationships among sampled families of Ditrysia, comparing working hypothesis to results of five-gene analyses**. Fig. 2A. Relationships among sampled families (only) according to composite working hypothesis [5]. Figs. 2B-F: Family relationships according to five-gene analyses, condensed from corresponding 123-taxon trees. Black triangles represent multiple exemplars. Bootstrap values (ML analyses) or posterior probabilities (Bayesian analysis) ≥50% are shown above branches. The corresponding 123- taxon trees, with support levels, are given in Additional files 3, 4, 5, 6 and 7. Fig. 2B. Nt123 ML analysis. Fig. 2C. Nt123 Majority rule consensus tree from Bayesian analysis; "§' symbol marks differences from nt123 ML tree. Fig. 2D. NoLRall2 + nt2 ML analysis. Fig. 2E. Nt12 ML analysis. Fig. 2F. Nt3 ML analysis.

**Figure 3:**
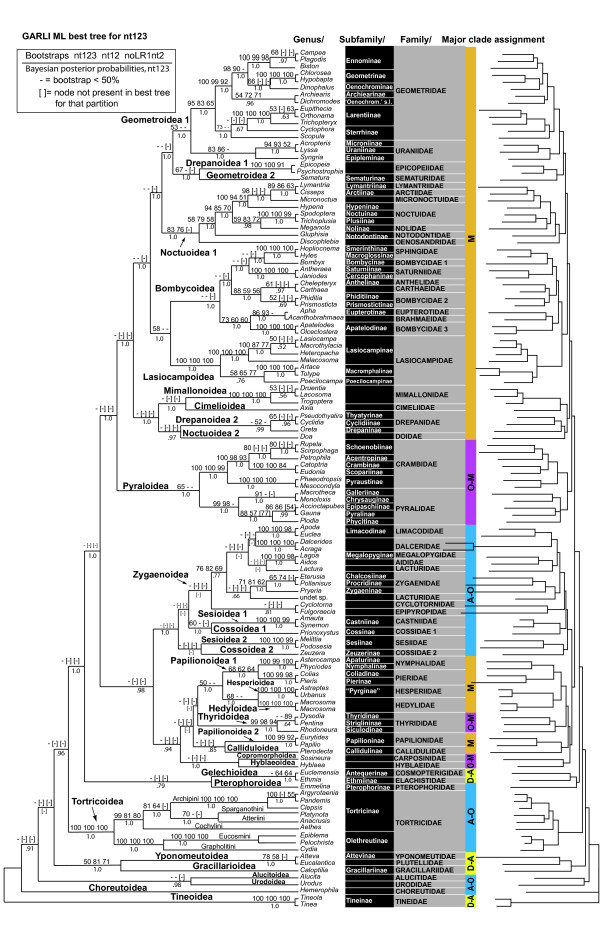
**Best 123-taxon ML tree found for nt123**. The ML nt123 topology is shown, with bootstrap values (BP) above branches (cladogram on left) separately calculated for ML nt123, ML nt12, and NoLRall2 + nt2, posterior probabilities from Bayesian nt123 analysis below branches. Dashes denote BP < 50%; brackets around BP or posterior probability mean group not recovered in the best ML tree for that character set and analysis. Branch lengths of the phylogram (right side) are proportional to total nucleotide change in ML nt123 tree. Major clade assignment (column to right of taxon names) according to working hypothesis (Fig. 2A): M = Macrolepidoptera; O-M = non-macrolepidopteran Obtectomera; A-O = non-obtectomeran Apoditrysia; D-A= non-apoditrysian Ditrysia.

The best trees found in the ML and Bayesian analyses are shown in condensed form in Fig. 2(B-F), with each terminal taxon representing a family or fraction thereof that proved monophyletic. Support values are shown on the branches. The Bayesian tree shown is a majority rule consensus of the trees sampled from the Markov chain process. For comparison, the expected relationships among (only) these families under the composite working hypothesis [1] are shown in Fig. 2A. The complete 123-taxon ML tree for nt123, together with bootstrap values for nt123, nt12 and noLRall2 + nt2, as well as Bayesian posterior probabilities for nt123, is shown in Fig. 3. Full ML trees in phylogram format, and bootstrap majority rule consensus trees, are presented for all data sets in Additional files 3, 5, 6, and 7. The full 123-taxon majority-rule consensus of trees sampled during the Bayesian analysis, with associated posterior node probabilities, is given in Additional file 5.

The ML tree and the Bayesian consensus tree for nt123 were largely identical, with just five discordant nodes (marked by §in Fig. 2C). The ML trees for noLRall2 + nt2 and nt12 were quite similar to each other, and only slightly less so to the nt123 topologies. The nt3 topology, in contrast, differs markedly from all the others, and will be discussed in a subsequent section. For the ML analyses apart from nt3, bootstrap support levels are generally high near the tips of the trees, typically within and among closely related families, whereas support at deeper levels - among superfamilies or more inclusive clades - is very weak, with almost no nodes reaching BP of even 50%. In striking contrast, support at deeper levels in the Bayesian analysis is very high, with posterior probabilities along the "backbone" of the tree nearly all 1.0; support at shallower levels is, if anything, weaker.

Comparison of the molecular trees (apart from ML nt3) to the morphology-based working hypothesis (Fig. 2A) can be summarized as follows, proceeding from more-inclusive to less-inclusive groupings. On the broadest scale, there is rough agreement with division of Ditrysia into four successively nested clades [6], but with major exceptions. In all analyses there is a clade consisting of most but not all of the superfamilies of Minet's Macrolepidoptera, with membership varying slightly among data sets. For nt123, this clade includes Geometroidea, Bombycoidea, Lasiocampoidea, and Noctuoidea, as well as Mimallonoidea, Drepanoidea and Cimelioidea (= Axioidea); placement of the last three is variable for noLRall2 + nt2 and nt12. In a substantial departure from expectation, however, the Pyraloidea, not traditionally considered Macrolepidoptera, are always grouped with these core macrolepidopterans, whereas the butterflies sensu Scoble [51] (Hedyloidea, Hesperioidea, Papilionoidea) are always more distantly related. The exact position of the butterflies sensu Scoble varies considerably among data sets, but they are always separated from core Macrolepidoptera + Pyraloidea by at least one lineage containing non-Macrolepidoptera. The remaining macrolepidopteran was the single representative of Calliduloidea, which was variably placed but never grouped with core macrolepidopterans, though it sometimes grouped within butterflies (nt123).

The other hypothesized major clades are also at best partially congruent with the molecular trees. The Obtectomera are never monophyletic, both because of the variable position of the butterflies sensu Scoble, and because each of the non-macrolepidopteran obtectomerans apart from Pyraloidea, i.e., Thyrididae, Hyblaeidae and Carposinidae, is quite unstably placed, sometimes grouping with non-obtectomerans. However, these last three groups are also allied often (always, in the case of Hyblaeidae) with either core Macrolepidoptera or butterflies, and in the nt12 analysis, all Obtectomera except Thyrididae and Callidulidae form a monophyletic group. In contrast, even near-monophyly is never supported for Apoditrysia, as the two non-apoditrysian groups Yponomeutoidea + Gracillarioidea and Gelechioidea are always nested (separately) within lineages that consist otherwise only of apoditrysians.

Support is similarly variable for two smaller-scale proposed groupings of superfamilies (Fig. 2A) [6]. The hypothesized clade consisting of butterflies sensu Scoble plus Geometroidea, Drepanoidea, Cimelioidea (= Axioidea) and Calliduloidea is never close to monophyletic; indeed, no subset of even two of these superfamilies is consistently grouped together. In contrast, the proposed grouping of Zygaenoidea + Sesioidea + Cossoidea is recovered fully by nt123, and is at least paraphyletic in trees for other data sets, although the predicted relationship among these superfamilies (Zygaenoidea (Cossoidea + Sesioidea)) was never seen. The butterflies sensu Scoble [51] were monophyletic for data sets excluding nt3. However, the widely accepted relationships among the superfamilies (Hedyloidea (Hesperioidea+ Papilionoidea)) were never recovered; the hedyloids always grouped with the hesperioids, and these with Nymphalidae + Pieridae, to the exclusion of Papilionidae, rendering Papilionoidea non-monophyletic. Monophyly of the "bombycoid complex" [52] is not supported, as Mimallonoidea never group with Bombycoidea + Lasiocampoidea. Only a single relationship among superfamilies received even moderately strong support in this study: the lone representative of Gracillarioidea was invariably grouped with the two Yponomeutoidea, with bootstrap support as high as 79% (for nt12).

The ML bootstrap support for nodes corroborating or contradicting the broad groupings just discussed is typically very weak (<< 50%). More support is evident, and congruence with previous hypotheses greater, for groupings at or near the superfamily level. Nine previously defined superfamilies were represented in our sample by more than one family. Monophyly was recovered in full for Pyraloidea and Gelechioidea in all analyses, with bootstraps sometimes > 60%, and for Bombycoidea in nearly all analyses, with weaker support. In three other cases, monophyly was consistently supported for a group consisting of at least a significant fraction of the families including the nominate family, and/or a plausible re-circumscription of the superfamily, with bootstrap support most often > 50% and sometimes > 80%. Geometroidea, if re-defined to include Epicopeiidae (included in Drepanoidea by Minet) were always monophyletic, though never with BP ≥ 50%. A core subset of Zygaenoidea (Aididae, Dalceridae, Lacturidae, Limacodidae, Megalopygidae, Zygaenidae), excluding only the enigmatic, highly divergent, parasitic families Epipyropidae and Cyclotornidae, was always monophyletic, sometimes with strong support (BP > 80%); Zygaenoidea as a whole are monophyletic in the ML nt123 analysis, albeit with weak support. Noctuoidea were always monophyletic, with moderate to very strong support (BP = 74-99%), except that *Doa *(Doidae), by far the longest-branched noctuoid exemplar, was never included. *Doa *grouped weakly instead with Cimeliidae (= Axiidae) or Drepanidae. This result was so surprising that we re-checked the identity of our *Doa *specimen, and re-extracted and partially re-sequenced it, to rule out laboratory error. Monophyly or near-monophyly was always lacking for three superfamilies, including Drepanoidea as discussed above. The two families of Sesioidea, Sesiidae and Castniidae, varied in position but always grouped separately with other taxa, never strongly but sometimes with BP > 50%. Papilionoidea, as we have seen, were always paraphyletic with respect to both Hedylidae and Hesperiidae.

The strongest resolution provided by our data lies within superfamilies and families. Most multiply-sampled families are recovered as monophyletic by noLRall2 + nt2, nt12 and nt123, often with very strong BP support. Apart from Noctuidae and Bombycidae, both of which have been previously shown to be non-monophyletic [14,53,54], only Cossidae are consistently non-monophyletic for all three data sets, albeit without even modest contrary bootstrap support. The nt123 analysis also fails to recover Lacturidae. Within several of the families and superfamilies that we sampled most extensively, including Geometridae, Pyraloidea, Noctuoidea and Tortricoidea, our results provide some of the first strong tests of hypothesized relationships. These findings are presented in detail in the Discussion.

### Tests for significance of non-monophyly

The results of the Approximately Unbiased tests [45] for significance of non-monophyly, carried out on eight previously hypothesized higher taxa that were never recovered in our trees, are presented in Table 1. This test is especially useful for assessing the confidence we can place in conclusions based on partial resolution of relationships, in cases such as the present one, where the data do not unambiguously resolve most or all individual nodes on the tree. By this test, monophyly can be confidently rejected for the hypothesized clade consisting of butterflies sensu Scoble [51] + Geometroidea + Drepanoidea + Cimelioidea + Calliduloidea [6], as *P *is less than 0.005 for all three character sets. Monophyly of Macrolepidoptera is rejected significantly by nt12 (*P *= 0.020), but not by either noLRall2 +nt2 (*P *= 0.106) or nt123 (*P *= 0.183). Strongly suggestive evidence is also seen for rejection of Drepanoidea as currently defined (*P *= 0.077). For the remaining five predicted groups, the data do not argue strongly against monophyly.

### Pairwise differences and rates of substitution

Uncorrected pairwise differences among all 123 species, an approximate indicator of the prevalence of multiple substitutions ("saturation"), are mainly in the range of 50-58% for nt3, while those at noLRall2 + nt2 are in the range of 3-8% (data not shown). Nt3 accounts for about 90% of the total character change in the nt123 data set under likelihood analysis, despite comprising only 33% of the total characters (Table 2). In contrast, noLRall2 + nt2 contributes only about 9% of total character change despite comprising 58% of all characters. In addition to nt3, the LRall2 character set (nt1 sites in codons specifying leucine or arginine in two or more taxa) also can undergo synonymous change; its average rate of substitution is quadruple that of the remainder of nt1 characters (noLRall2), in which synonymous change cannot be detected.

**Table 2:** Comparison of tree statistics among character sets^a^

	A	B	A × B
**Character set**	**Number of characters**	**Ave. Δ/character**	**Tree length**

**nt123**	6,633(= 1.0)	35.17	233,312 (= 1.00)

**nt12**	4,422(0.67)	8.76	38,751 (0.17)

**nt3**	2,211(0.33)	99.79	220,645 (0.95)

**nt2**	2,211(0.33)	5.63	12,452 (0.05)

**nt1**	2,221(0.33)	15.64	34,579 (0.15)

**noLRall2**	1,631(0.25)	6.57	10,723 (0.05)

**LRall2**	580(0.09)	26.67	15,469 (0.07)

**noLRall2 + nt2**	3,842(0.58)	5.54	21,283 (0.09)

^a ^Based on likelihood analyses of 5-gene, 123-taxon character sets and subsets. The single constraint tree on which parameters are optimized is derived from analysis of nt123.

A, number of characters; B, average number of substitutions per character across all branches; A × B, Tree length = number of substitutions for all characters across all branches. Numbers in parentheses represent comparisons to the nt123 values, defined as 1.0.

### Nucleotide composition heterogeneity and its phylogenetic consequences

The results of chi-square tests for compositional heterogeneity are shown in Table 3. Neither for all taxa nor for any of the 13 taxon subsets could homogeneity be rejected for the noLRall2 + nt2 character set. Even with invariant characters (62% of total) removed, only Zygaenoidea were significantly non-homogeneous, due in part to the highly divergent taxon Epipyropidae. In contrast, nt3 showed highly significant heterogeneity across all 123 taxa and within all 13 taxon subsets as well.

**Table 3:** Results of Chi square tests of nucleotide composition homogeneity.

*P *value for character set			
**Taxon (number of species)**	**noLRall2 + nt2**	**noLRall2 + nt2 (variable sites only)**	**nt3**

all taxa (123)	>0.999	>0.999	<0.001

Bombycoidea (13)	>0.999	0.995	<0.001

Lasiocampidae (7)	>0.999	0.338	<0.001

Geometridae (13)	>0.999	0.922	<0.001

Noctuoidea no Doidae (9)	>0.999	>0.999	<0.001

Papilionoidea(10)	>0.999	0.421	<0.001

Tortricidae (9)	>0.999	>0.999	<0.001

Pyraloidea (12)	>0.999	>0.999	<0.001

Crambidae (7)	>0.999	>0.999	<0.001

Pyralidae (5)	>0.999	>0.999	<0.001^a^

Zygaenoidea (13)	0.699	0.010	<0.001

Zygaenoidea, no Cyclotornidae, no Epipyropidae (11)	0.816	0.043	<0.001

Zygaenoidea, no Cyclotornidae (12)	0.561	0.004	<0.001

Zygaenoidea, no Epipyropidae (12)	0.900	0.075	<0.001

number of characters:	3842	1467	2211

Compositional heterogeneity in nt3 may account for some of the unusual properties of the ML topology derived from nt3 alone (Fig. 2F). That tree, as noted earlier, differs strikingly from those for all other character sets, and fails to recover a number of groupings strongly supported both by other character sets and by morphology. For example, it breaks up the strongly-supported families Geometridae, Uraniidae and Zygaenidae. The nt3 tree also has markedly lower support levels than those for other data sets. Despite providing about 90% of the total character change, the nt3 character set alone yields bootstrap support > 50% for only about half as many nodes (25) as does the full data set (nt123; 48 supported nodes), fewer even than the noLRall2 + nt2 character set (32 supported nodes).

More direct evidence that compositional heterogeneity can affect trees based on nt3 alone comes from comparison of the NJ trees computed from LogDet distances, which correct for compositional heterogeneity, to those computed on ML distances on the GTR + G model, which do not (trees not shown). For nt3, these two trees are very different from each other, sharing only 51 of a possible 120 nodes. For noLRall2 + nt2, in contrast, they are quite similar, sharing 99 of 120 nodes. The case for an influence of composition is further supported by the Neighbor Joining analysis of pairwise Euclidean distances computed from just the proportions of the four nucleotides in each taxon (Additional file 8). The range of the compositional distances themselves (largest minus smallest) is about 4.5 times greater for nt3 than for noLRall2 + nt2. The tree for noLRall2 + nt2 tree is mostly comb-like with short branches, consistent with prevailing compositional homogeneity. The few distinct clusters, reflecting shared deviation from typical composition, appear to group taxa that are missing a relatively high proportion of data, frequently in the same gene regions. Thus, such compositional heterogeneity as is evident in noLRall2 + nt2 is apparently due to intra- and/or inter-genic heterogeneity coupled with differential sampling of those regions. None of the obviously incorrect groupings (i.e., those contradicting very strong previous evidence) based on composition alone are also found in likelihood or distance analysis based on standard substitution models. The results of the corresponding analyses of nt123 are very similar, providing likewise little evidence for an influence of composition that contradicts very strong previous evidence.

The results for nt3 alone are very different. Here, groupings in the composition-based NJ tree appear to be largely independent of missing data, reflecting instead real differences in mean composition among taxa. In several instances, groups based only on composition match clearly incorrect groupings seen in the ML tree and the NJ tree on ML distances (see Additional files 8, 9). For example, the most derived group in the nt3 NJ tree based on composition pairs one species of Uraniidae with one species of Geometridae, and these together with Tortricidae. Similar though not identical groupings are seen in the NJ tree on GTR distances, reaching bootstrap support as high as 77% and 78%, and also in the ML tree of Fig. 2F. Thus, it appears that in the case of nt3 alone, sufficiently aberrant composition can sometimes overwhelm true phylogenetic signal.

## Discussion

In this section we first review the implications of our findings for the analysis of molecular data on deep-level lepidopteran relationships. We then review the bearing of our results on current understanding of those relationships themselves.

### Heuristic search efficiency and computational effort

In preliminary analyses prior to those described here we had repeated each GARLI run 20 times (20), a typical number in applications of this program so far [14,29,30]. Surprising discrepancies between the initial tree estimates for different character sets prompted us to wonder if these were really the best available estimates. The ensuing, much more extensive tree searches reported here revealed, first, better trees for all data sets. Moreover, for all data sets except nt3, the same best topology was found many times (114-925, or 1.1-9.3%) in 10,000 searches, making it plausible, though not provable, that we actually found the global ML topology. (For nt3, for which the best topology appeared only twice, we are less likely to have found the global ML tree.) Also revealed by these searches were (to us) surprisingly large sets of near-best trees, with only slightly lower likelihood scores, yet including topologies strikingly different from the best tree, underscoring the limited resolving power of our data.

We tentatively conclude from these results that GARLI analyses of data sets the size of ours should routinely include not just tens of searches but hundreds, if one wants to be confident of having the best feasibly obtainable tree. But, given that the differences between our initial trees and the better ones found subsequently are very weakly supported by any measure (see below), is the improvement worth the large additional computational effort? The answer is often likely to be yes, for at least two reasons. First, some types of hypothesis tests for which tree estimates are used do not explicitly take topological uncertainty directly into account, thereby placing a premium on the accuracy of the input tree. For example, in our study, the improved tree estimates generally raised the *P *values of the significance tests for non-monophyly of previously-proposed groups, as compared to those based on the initial trees found using just 20 GARLI searches (data not shown). In 16 total comparisons, the new *P *value was equal to the initial one in two, moderately to substantially larger in ten, and smaller (slightly) in just four. These differences were caused specifically by change in the unconstrained tree estimates.

A second reason to value even small, hard-won improvements in likelihood score is that some problems, including ditrysian phylogeny, may be difficult enough to resist strong resolution by any one data source for a long time to come. In the interim, the most convincing means of favoring one hypothesis over another may be congruence among multiple data sets, each providing only weak support by itself. Thus, credibility is lent to the phylogeny estimates presented here, despite their low support levels, by the fact that very similar relationships among lepidopteran superfamilies are emerging from an independent molecular study using a different but comparable gene sample, and a larger taxon sample (M. Mutanen, L. Kaila, N. Wahlberg, personal communication).

### Support levels and possible reasons for low bootstrap support at deeper levels

The overall pattern of bootstrap support in our ML analyses is that families and divergences within them are generally strongly supported; superfamilies and divergences within them are only sometimes strongly supported; and relationships among superfamilies almost always have very weak support, with bootstraps often < 20% (see Additional files 4 and 7).

Why is support along the "backbone" so low? There are several possibilities. First, given the extensive search needed to find the best feasibly-obtainable ML trees, low ML bootstrap support at deeper levels might be thought to reflect insufficient effort - a single GARLI search - on each pseudo-replicate. Search effort surely has some effect on bootstrap efforts, but we doubt that it is the main explanation. The literature on per-replicate search effort required for accurate bootstrap percentage estimation [55,56], while limited thus far to parsimony analyses, suggests that a plateau in mean BP is quickly reached as one increases heuristic search effort from simplified fast methods to somewhat more elaborate methods (e.g. limited branch swapping) to full standard search methods (e.g., those incorporating extensive branch swapping). It further suggests that the plausible prediction of increased BP with more thorough searches is realized mainly for BP values which were low to begin with. Preliminary experiments included in our initial analyses (data not shown) point in the same direction: very low initial BP values sometimes increased substantially (up to 20-30% in absolute value) with increased search effort per pseudo-replicate, but never reached moderate or strong levels (≥ 70%); initially high BP values (≥ 80%) changed little with increased pseudo-replicate search effort.

Strong conflict among genes is a second possible explanation for low BP values at deeper levels, but in our data set, the rare instances of such conflict are restricted to within-family relationships (see Additional file 3). Support might have been higher had our ML analysis modeled synonymous and non-synonymous character sets separately, but the near-identical topologies produced by our unpartitioned ML and partitioned Bayesian analyses for all nucleotides suggests that the effect would not be dramatic. The most plausible explanation for low support is that the branches along the "backbone" are very short, as evidenced in the phylogram of Fig. 3, in contrast to the very long branches subtending some superfamilies (e.g., Tortricoidea, Lasiocampoidea, Hesperioidea) and/or subgroups therein. Short internodes along the "backbone", which may reflect rapid radiation, suggest that very large amounts of sequence, as well as more accurate modeling of character change, will be needed to firmly resolve these nodes.

In contrast to low support under ML, posterior probabilities along the "backbone" in the Bayesian analyses were very high, mostly 1.0. The sole purpose of our Bayesian analysis was to examine the effect of partitioned analysis on tree topology. The interpretation of the associated posterior probabilities is problematic, due to their often-reported tendency toward "overcredibility" (e.g. [41,57,58]). Lewis et al. [59] attribute "overcredibility" to the failure of the tree-proposal step in current Bayesian phylogenetic algorithms to allow the possibility of polytomies. In the absence of true signal, this restriction can artificially confer very high posterior probabilities on arbitrary resolutions. This explanation seems quite consistent with our findings.

### Differing properties among character sets and their implications

Several potential benefits motivate our focus on separating and independently analyzing sites undergoing synonymous versus non-synonymous change, as exemplified by our distinction between the character sets "noLRall2 + nt2" and "LRall2 + nt3". These categories can be defined in all protein-coding sequences, and the substitutions they undergo are known to follow markedly different rules. To some extent, then, they provide independent lines of phylogenetic evidence, thereby boosting our confidence in groupings that they separately recover. Separate analysis also allows us to discover the evolutionary properties peculiar to each, and to account for these when considering the two character sets together. Thus, it is reassuring that although there are numerous differences in detail involving nodes with little support, particularly at deeper levels, trees based on noLR + nt2, nt12 and nt123, which though not fully independent span a gradient from entirely non-synonymous to predominantly synonymous evolution, are quite similar overall. They are generally concordant for nodes with modest to strong BP support. Even moderately strong conflict - reciprocal BP ≥ 70% support for incompatible groupings - is essentially absent.

Analysis of nt3 alone, however, complicates the picture. Despite contributing about 90% of the total evolutionary change for nt123, nt3 by itself provides relatively weak resolution, and fails to recover many well-established nodes. Yet when added to nt1 + 2, it can often greatly increase support for those nodes (e.g. Geometridae, Pyraloidea, Yponomeutidae), particularly at shallower levels, providing dramatic examples of "hidden support" [60]. On the other hand, adding nt3 sometimes markedly *decreases *support for deeper nodes, e.g. Gelechioidea, "core" Zygaenoidea, Yponomeutoidea + Gracillarioidea. Nt3 seems to contain a complex mixture of true phylogenetic signal and conflicting signal from non-phylogenetic sources. The latter undoubtedly stems in part from non-homogeneity of base composition. It appears that for shallower divergences the non-phylogenetic signal in nt3 is relatively easily overcome by the addition of non-synonymous signal. For deeper divergences, in contrast, it appears that either true phylogenetic signal at nt3 is weakened by saturation, or non-phylogenetic signal becomes relatively stronger, or both, leading typically to less resolving power. And yet, nt3 does carry some true signal for deeper divergences, possibly because it undergoes at least some non-synonymous change. For example, it is probably not coincidence that only with nt3 included do we completely recover, albeit with weak support, both Zygaenoidea and Sesioidea + Cossoidea + Zygaenoidea. Our analyses of nt3 alone provide one of the first examples of an influence of compositional heterogeneity on estimated phylogeny at relatively low taxonomic levels; previous demonstrations have mostly involved much deeper divergences (but see Gruber et al. [61]). One might take comfort in the disappearance of the obvious effects of composition on topology when nt3 is combined with other character sets. Compositional heterogeneity remains a likely contributor, however, to the instability and lower support that inclusion of nt3 brings to some deeper-level groupings. The problem cannot be easily dismissed.

Given that different character sets can differentially support, and/or obscure, each individual node, treating all character sets as belonging to a single population of characters (as in our nt123 ML analysis) is clearly not the most effective way of extracting phylogenetic information from the data set. Ideally, one would analyze all character sets simultaneously, using a model that fully accounted for the differences in evolutionary behavior among them. The widely-available methods for partitioned analyses, however, do not yet include correction for heterogeneity of nucleotide composition, a key point of difference between mainly synonymous and mainly non-synonymous character sets. In the review below, we therefore adopt an interim strategy for assessing progress on ditrysian phylogeny: a group is considered to be supported by the data set as a whole to the degree that it is (a) strongly supported by one or more character sets, and (b) at most weakly contradicted by others. At present, no single analysis can tell the whole story.

### Current understanding of ditrysian phylogeny

In this section we ask, how much progress did this exploratory study yield toward a robust phylogeny estimate for Ditrysia, and toward testing the working hypotheses compiled by Kristensen [5]?

The near-total lack of strong support for nodes subtending multiple superfamilies, with especially low bootstraps along the "backbone", is sobering. We expected more from 6.7 kb of sequence data chosen specifically for their suitability for addressing this problem. It appears that robust node-by-node resolution of among-superfamily ditrysian relationships will require several to many times as much sequence as analyzed here, in addition to expanded taxon sampling, particularly among the non-obtectomeran lineages. Fortunately, two independent efforts to provide such additional data are underway (see http://www.Leptree.net).

Low bootstraps for deep nodes notwithstanding, however, our current data do provide important first steps toward resolving ditrysian phylogeny. How can this be? Conventional bootstrap values can greatly underestimate the amount of structure present in a large, noisy data set, because they take into account only nodes that agree completely between pseudo-replicate trees, ignoring partial agreement on those groupings [62]. Thus, given the taxon sample size, we think that the approximate overall concordance of our trees with the "backbone" hypothesis in Fig. 2A is unlikely to be accidental, despite the lack of bootstrap support. Our results provide some of the first quantitative phylogenetic evidence for broad subdivisions of Ditrysia resembling those of Minet [6], albeit with important differences. The clearest point of correspondence is that all analyses apart from nt3 yield a clade consisting of most Macrolepidoptera plus one or more non-macrolepidopteran Obtectomera, and excluding all non-obtectomerans.

This mostly macrolepidopteran clade, however, also harbors one of the strongest departures from the working hypothesis. The Pyraloidea, traditionally considered non-macrolepidopterans, invariably group with the "core" Macrolepidoptera identified here (which exclude butterflies), while the butterflies sensu Scoble, always traditionally considered macrolepidopterans, never do so. Despite weak support for individual nodes, the Approximately Unbiased test [45] provides statistical evidence against monophyly of the Macrolepidoptera as previously defined (Fig. 2A), significantly rejecting it for nt12 (*P *= 0.02) although not for the other character sets. Minet's [6] exclusion of Pyraloidea from Macrolepidoptera was based on their supposed lack of his synapomorphy 17, a feature of the base of the forewing. Recent unpublished observations by one of us (MAS), however, strongly suggest that this feature is in fact characteristic of pyraloids. The distribution of this trait deserves further study in other superfamilies as well.

The existence of a clade comprising "core Macrolepidoptera" plus Pyraloidea, which we predict that future work will confirm, is likely to prompt re-examination of hypotheses about the evolution of the thoracic or abdominal ultrasound-detecting "ears" that characterize the superfamilies Noctuoidea, Geometroidea, Drepanoidea and Pyraloidea. These together contain over 90% of species in the putative clade. The "ear" found in each superfamily shows a unique location and anatomy, and has been thought to represent an independent origin. Our result prompts contemplation, at least, of the possibility of fewer origins, conceivably even a single origin in the common ancestor of the proposed clade (though there is disagreement among the authors of this work regarding the plausibility of this hypothesis). This alternative hypothesis would require only a few independent losses of the "ear," in the ancestors of Bombycoidea + Lasiocampidae, Cimeliidae (= Axiidae), and Sematuridae + Epicopeiidae. The observation [9] that the anatomy of the "ear" and/or the location of its opening can vary between sister families (Pyralidae versus Crambidae) or between sexes within the same family (Uraniidae) suggests that transformation among widely differing types of "ear" is at least plausible.

The unexpected position of the butterflies is also reflected in the strongest result from our tests for non-monophyly, namely the decisive rejection (*P *< 0.005, all character sets) of the proposed clade aligning butterflies and allies with Geometroidea, Drepanoidea, Cimelioidea (= Axioidea) and Calliduloidea [6]. In our trees, no two of these taxa consistently group with or even near each other. It seems safe to abandon this conjecture. An alternative hypothesis about phylogeny of the major macrolepidopteran groups, grouping Geometroidea with Noctuoidea and these together with Bombycoidea + Lasiocampoidea, is worthy of contemplation because it is supported, albeit weakly, in all our analyses. In contrast, placement of the several small, morphologically isolated and highly divergent macrolepidopteran superfamilies may be very difficult. The problem is illustrated by the unstable position of Mimallonidae and our inability to significantly reject their alliance with Bombycoidea and Lasiocampoidea, despite their failure to ever group near these superfamilies.

Within several large superfamilies of the "core Macrolepidoptera + Pyraloidea" clade, our results provide some of the first strong tests of hypothesized relationships (Figs. 2, 3). In Geometroidea, our findings corroborate, albeit with weak support, the grouping of Sematuridae, which lack abdominal tympanal organs, with Uraniidae and Geometridae, which possess them. A novel result is that all data sets place Epicopeiidae, included in Drepanoidea by the working hypothesis, either next to or within Geometroidea. The strongest signal comes from nt123, which resolves Epicopeiidae as sister group to Sematuridae (BP = 67%), with which they share the lack of tympanal organs. Within Geometridae, one of the largest families of Lepidoptera, we sampled all subfamilies, finding moderate to very strong support for nearly all relationships among these (Fig. 3), and strong agreement with groupings seen in other recent molecular studies of this family [21,63].

In Noctuoidea, our findings very strongly (BP = 94-100%) corroborate previous morphological and/or molecular evidence for monophyly of: (a) the quadrifid forewing clade of families; (b) within this, the clade of "trifine" hindwing subfamilies; and (c) a clade comprising most quadrifine hindwing Noctuidae plus Arctiidae and Lymantriidae, excluding Nolidae (the "LAQ" clade of Mitchell et al. [53]), *contra *recent morphology-based hypotheses [64]. The recently erected family Micronoctuidae [64] also appears to fall in the "LAQ" clade. The most surprising result is the failure of *Doa *(Doidae) to group with the remaining Noctuoidea, despite its possession of the very distinctive morphological synapomorphies of the superfamily, including a metathoracic tympanal organ and two MD setae on larval T3 [65]. No position for *Doa *is strongly supported, however, and noctuoid monophyly is not significantly rejected by the Approximately Unbiased test. Thus, it remains possible that *Doa *will group with other noctuoids upon further gene and taxon sampling. The postulated sister group relationship between Doidae and Notodontidae [65], on the other hand, now seems very unlikely, given the strong support (BP = 83%, nt123) for a clade comprising all sampled Noctuoidea except *Doa*.

Pyraloidea are recovered by all data sets (except nt3), albeit with low support (BP = 65%, nt123). Though our sampling of subfamilies is incomplete, the five genes appear to offer strong resolution of relationships within this superfamily, including very strong bootstrap support (BP ≥ 99%) for monophyly of both Pyralidae and Crambidae as sampled. Divergences among all exemplars of Pyralidae, representing all five subfamilies, were strongly resolved (BP 80-100%). Relationships among subfamilies nearly match a previous morphology-based tree [66], requiring only a trade in position of the subfamilies Pyralinae and Phycitinae. In Crambidae, relationships of the five (of 14) subfamilies sampled were also strongly resolved, corresponding well to the morphological hypothesis of Yoshiyasu [67], less well to that of Solis and Maes [16].

In contrast to their success in the foregoing clades, our data do not strongly resolve relationships of the butterflies sensu Scoble [51]. The three superfamilies do form a clade, but only in analyses excluding nt3, and neither the monophyly of Papilionoidea, nor any of the accepted relationships among the families thereof except the basal position of Papilionidae, is supported by any analysis. On the other hand, the Approximately Unbiased test (Table 1) does not reject monophyly for Papilionoidea (P > 0.38), and bootstrap supports are mostly low for groupings contradicting expectation, the highest being 68% for the unexpected pairings of Hedylidae with Hesperiidae and Pieridae with Nymphalidae. Thus, apart from qualitatively corroborating Scoble's grouping of *Macrosoma *(Hedylidae) with butterflies rather than Geometridae, we are unable to strongly confirm or refute any previous hypothesis about butterfly relationships. Our results raise the possibility, however, that butterfly relationships will undergo significant revision as more data accumulate.

Evidence both for and against predicted relationships is less strong in the lower ditrysian lineages than in Obtectomera, which were much more extensively sampled. Nonetheless, some tentative conclusions emerge. Two non-apoditrysian lineages are always nested well inside the Apoditrysia as currently defined, the monophyly of which is consequently never supported. The pairing of Yponomeutoidea and Gracillarioidea, a novel grouping so far as we are aware, is the most strongly supported among-superfamily relationship in our study (BP = 79%, nt12).

Within the lower Apoditrysia ("A-O" in Fig. 3), one of the few previous postulates of among-superfamily relationships [6] groups Zygaenoidea with Sesioidea + Cossoidea. Agreement between our analyses and previous hypotheses is ambiguous throughout this putative clade, suggesting signal too weak to be decisive. The proposed groupings are only sometimes monophyletic, and yet never strongly contradicted. The trio of superfamilies is fully recovered by nt123 (Fig. 2B), albeit with very weak support, and is at least somewhat coherent in the other analyses. In the nt12 tree (Fig. 2E), for example, it is basal and paraphyletic with respect to all other taxa except Tineidae. Thus, the proposed grouping Sesioidea + Cossoidea is never monophyletic, but in nt123 it is at least paraphyletic, comprising the two lineages most closely related to Zygaenoidea. Within this assemblage, however, neither Sesioidea (Sesiidae + Castniidae) nor Cossidae are monophyletic in any analysis.

Our data provide similarly qualified support for Zygaenoidea, the monophyly of which is uncertainly established by morphology [68]. The eight families sampled (of 12) were grouped together by one data set, nt123, but with weak support. We did however find a consistently monophyletic core group of six zygaenoid families, with bootstrap support as high as 82% (nt12, Fig. 2E). Excluded from the "core Zygaenoidea" were the parasitic families Epipyropidae and Cyclotornidae. The weakly-supported sister group relationship between these families seen in nt123 (Fig. 2B) seems credible, despite the exceptionally long branches subtending both, because of their bizarre shared larval habit of ectoparasitism on Auchenorrhyncha. Within the "core Zygaenoidea" we find qualified support for the morphologically-defined "limacodid group" of families [68], here represented by Limacodidae, Dalceridae, Megalopygidae and Aididae. These form a clade, weakly supported, in the noLRall2 + nt2 tree, and a paraphyletic group in other analyses. Relationships within the "limacodid group" agree partially with the morphological cladogram of Epstein [69], in that Dalceridae are most often grouped with Limacodidae (nt123, noLRall2 + nt2), albeit with weak support. The only strongly supported node, however, unites Aididae with Megalopygidae (BP ≥ 99%), in which they were formerly included, *contra *Epstein's hypothesis grouping them with Dalceridae + Limacodidae. Within Zygaenidae our data moderately to strongly support the relationships reported by Niehuis et al. [70] for the three subfamilies sampled.

In Tortricoidea, finally, though our sampling is limited to two of three subfamilies and six of 21 tribes, the five genes appear able to offer decisive resolution, as all nodes are strongly supported. Support is 99-100% for monophyly of the two subfamilies as thus far sampled. The representative of Cochylini, in the past treated as a separate family [71,72], was strongly placed as sister group to the two other sampled tribes of Tortricinae, consistent with the proposal of Kuznetsov and Stekolnikov [73,74].

## Conclusion

The five genes/6.7 kb and 123 species analyzed here provide sufficient information to: (a) corroborate the broad outlines of the current working phylogenetic hypothesis for Ditrysia; (b) conclusively demonstrate that several prominent features of that hypothesis, including the position of the butterflies, need revision; and, (c) strongly resolve the majority of family and subfamily relationships within superfamilies as thus far sampled. However, these data alone cannot strongly resolve node-by-node relationships among superfamilies of Ditrysia. Such resolution will clearly require a substantial increase in both sequence and taxon sampling. Moreover, given the complexity of character variation at deeper levels of divergence, especially the saturation and strong compositional heterogeneity characterizing nt3, full resolution of ditrysian relationships will require careful dissection of true from misleading phylogenetic signal.

## Abbreviations

BP: Bootstrap Percentage; GARLI: Genetic Algorithm for Rapid Likelihood Inference [33]; GTR + G + I: General Time Reversible substitution model with Gamma distribution correction for among-site rate variation and a separate class of Invariant sites; ML: Maximum Likelihood; NJ: Neighbor-joining tree-building algorithm; Nt1, nt2, nt3: codon position 1, codon position 2, codon position 3, respectively; NoLRall2: subset of nt2 sites (columns in the data matrix) which excludes all those occurring in codons which specify either leucine in two or more taxa, or alanine in two or more taxa; LRall2: complement of the preceding; a subset of nt2 sites which includes only those occurring in codons which specify either leucine in two or more taxa, or arginine in two or more taxa; RT-PCR: reverse transcription from mRNA; followed by DNA PCR.

## Authors' contributions

JB, JWB, SC, MPC, DRD, AH, AYK, CM, CP, JCR, AR, and SW participated in the design and coordination of the study. JBA, JB, SC, DRD, ME, WH, AH, DHJ, AYK, IJK, CM, MAS and SHY collected and identified specimens. JCR directed, and JCR and SC participated in, generation and alignment of the sequence data. MPC, AYK, JCR, AZ and ALB performed the phylogenetic analyses, which were led by AZ. MPC, CM, CP, JCR, AR, SW and AZ participated in drafting the manuscript, coordinated by CM, and all the authors participated in the editing of the manuscript. All the authors read and approved the final manuscript.

## Supplementary Material

Additional file 1 **Specimen information and Genbank numbers**. For each specimen sequenced we list superfamily, family, genus and species name, code name, collection locality, GenBank accession numbers for all sequences, and missing and partial sequences. Numbers after the superfamily name indicate number of families sampled/number of families total. An L in parentheses after the species name means the specimen sequenced was a larva; a P in parentheses means it was a pupa. All other specimens were adults. An 'X' in the Genbank number table means that no sequence was obtained for that gene in that taxon. Click here for file

Additional file 2 **Data matrix**. The final sequence alignment, excluding alignment-ambiguous regions, is presented in sequential Nexus format. The file includes charsets for different genes, nucleotides and LR/noLR. Taxon names are the code names given in Additional file 1. Click here for file

Additional file 3 **Single-gene bootstrap analyses**. We present a table of bootstrap values obtained from a separate analysis of all nucleotides for each gene, for all nodes on the all-nt ML tree plus all other nodes supported by BP of 50% or greater by any gene. We summarize the evidence on bootstrap-supported groupings that conflict with those found for other individual genes, with the all-gene result, and with conventional understanding of relationships, and examine the two instances of strong conflict. Click here for file

Additional file 5 **Nt123 Bayesian analysis, partitioned noLRall2 + nt2 vs. LR + nt3**. Majority rule consensus of trees sampled from partitioned Bayesian analysis of nt123 with partitions noLRall2 + nt2 versus LRall2 + nt3. Two runs, 10106 trees sampled from each, with standard deviation of split frequencies < 0.01. Click here for file

Additional file 6 **123-taxon ML tree & bootstrap for noLRall2 + nt2**. Part A: noLRall2 + nt2 best ML tree found in 10,000 replicate GARLI searches, GTR + G + I model, phylogram format. Part B: noLR2all + nt2 bootstrap majority rule consensus tree, generated in PAUP, from 1000 GARLI ML bootstrap replicates, GTR + G + I model. Click here for file

Additional file 7 **123-taxon ML tree & bootstrap consensus tree for nt12**. Part A: nt12 best ML tree found in 10,000 replicate GARLI searches, GTR + G + I model, phylogram format. Part B: nt12 bootstrap majority rule consensus tree, generated in PAUP, from 1000 GARLI ML bootstrap replicates, GTR + G + I model. Click here for file

Additional file 8 **Effects of compositional heterogeneity on inferred relationships, compared between nt3 and noLRall2 + nt2**. Part A. Analysis of variable nt3 characters. Part B. Analysis of noLRall2 + nt2 characters. Click here for file

Additional file 9 **123-taxon ML tree & bootstrap consensus tree for nt3**. Part A. nt3 best ML tree found in 10,000 replicate GARLI searches, GTR + G + I model, phylogram format. Part B. nt3, bootstrap 50% majority rule consensus tree, generated in PAUP, from 1000 GARLI ML bootstrap replicates, GTR + G + I model. Click here for file

Additional file 4 **123-taxon ML tree & bootstrap consensus tree for nt123**. Part A: nt123, best ML tree found in 10,000 replicate GARLI searches, GTR + G + I model, phylogram format. Part B: nt123, majority rule consensus tree from 1000 GARLI ML bootstrap replicates, generated in PAUP. Click here for file
