# Supplementary material for: Toward reconstructing the evolution of advanced moths and butterflies (Lepidoptera: Ditrysia): an initial molecular study
Source: BMC Evol Biol. 2009 Dec 2;9:280. doi: 10.1186/1471-2148-9-280 (PMC2796670; doi:10.1186/1471-2148-9-280)
Supplement: Additional file 3 — Single-gene bootstrap analyses. We present a table of bootstrap values obtained from a separate analysis of all nucleotides for each gene, for all nodes on the all-nt ML tree plus all other nodes supported by BP of 50% or greater by any gene. We summarize the evidence on bootstrap-supported groupings that conflict with those found for other individual genes, with the all-gene result, and with conventional understanding of relationships, and examine the two instances of strong conflict. [file 1471-2148-9-280-S3.DOC]

**Additional File 3 – Single–Gene Bootstrap Analyses**

To determine whether conflicting signal among genes might significantly complicate phylogenetic analysis, we conducted a maximum likelihood bootstrap analysis (1000 pseudo-replicates) for each gene separately (all nucleotides), using the default settings of GARLI. We scanned the results for groupings that conflicted strongly (bootstrap support of 70% or more) with those found in other individual gene results, with the all-gene result, or with conventional understanding of relationships. While we were looking specifically for real differences among gene trees, such conflicts could also result from mistakes in assembly, labeling, or homology assessment (e.g., paralogy). Thus, this analysis is also part of the error-checking process.

Below we present the results of the bootstrap analysis by means of a table and an accompanying all-nt tree. We then summarize the conflicts found, and examine more closely the two instances of strong conflict.

**Table A1 (next page).** The following table shows the bootstrap values obtained for each gene, for all nodes on the all-nt ML tree plus all other nodes supported by BP of 50% or greater by any gene. Only bootstraps of 50% or more are shown. The node numbers in the second and third columns correspond to node numbers on the version of the all-nt ML tree shown below the table. Groupings which conflict with a given node on the all-nt tree are marked by that node number followed by an ‘A’.

| Family of included terminal taxa | Node | Terminal taxa and/or internal nodes subtended | All 5 genes | CAD | DDC | Enolase | Period | Wingless |
| --- | --- | --- | --- | --- | --- | --- | --- | --- |
| Geometridae | 1 | *Campaea* + *Plagodis* | 68 | 88 |  | 52 |  |  |
| Geometridae | 1A | *Plagodis + Biston* |  |  |  |  |  | 58 |
| Geometridae | 2 | *Campaea* + *Plagodis* + *Biston* | 100 | 84 |  | 73 |  |  |
| Geometridae | 3 | *Chlorosea* + *Hypobapta* | 100 | 100 |  | 80 | 96 | 99 |
| Geometridae | 4 | 3 + *Dinophalus* | 66 | 89 |  |  |  |  |
|  | 5 | 2 + 4 | 98 | 67 |  | 72 |  |  |
| Geometridae | 6 | *Archaearis* + *Dichromodes* | 54 |  |  |  |  |  |
|  | 7 | 5 + 6 | 100 | 94 |  |  |  |  |
| Geometridae | 8 | *Eupithecia* + *Orthonama* | 53 |  |  |  | 52 |  |
| Geometridae | 8A | *Trichopterix* + *Orthonama* |  |  |  | 89 |  |  |
| Geometridae | 9 | *Eupithecia* + *Orthonama* + *Trichopterix* | 100 | 97 |  |  | 92 | 63 |
| Geometridae | 10 | 9 + *Cyclophora* |  |  |  |  |  |  |
| Geometridae | 10A | *Cyclophora + Scopula* |  | 66 |  |  |  |  |
| Geometridae | 11 | 10 + *Scopula* | 73 | 64 |  |  |  |  |
| Geometridae | 12 | 7 + 11 | 100 |  |  |  |  |  |
| Uraniidae | 13 | *Acropteris* + *Lyssa* | 94 |  |  |  |  | 94 |
| Uraniidae | 14 | 13 + *Syngria* | 83 | 70 |  |  |  |  |
|  | 15 | 12 + 14 | 53 |  |  |  |  |  |
| Epicopeidae | 16 | *Epicopeia* + *Psychostrophia* | 100 | 99 | 85 | 61 | 87 |  |
| Sematuridae | 17 | 16 + *Sematura* | 67 |  |  |  |  |  |
|  | 18 | 15 + 17 |  |  |  |  |  |  |
| Lymantriidae + Arctiidae | 19 | *Lymantria* + *Cisseps* | 89 |  |  |  |  |  |
|  | 20 | 19 + *Micronoctua* | 98 |  |  |  |  |  |
| Noctuidae | 21 | 20 + *Hypena* | 100 | 86 |  |  |  |  |
| Noctuidae | 22 | *Spodoptera + Trichoplusia* | 100 | 75 | 96 |  | 77 |  |
| Nolidae | 23 | 22 + *Meganola* | 59 | 82 |  |  |  |  |
|  | 24 | 21 + 23 | 94 | 86 |  |  |  |  |
| Notodontidae | 25 | 24 + *Gluphisia* | 58 | 76 |  |  |  |  |
| Oenosandridae | 26 | 25 + *Discophlebia* | 83 | 76 |  |  |  |  |
|  | 27 | 18 + 26 |  |  |  |  |  |  |
| Bombycoidea: Sphingidae | 28 | *Hopliocnema* + *Hyles* | 100 | 100 | 54 |  | 72 |  |
| Bombycidae 1 | 29 | 28 + *Bombyx* |  |  |  |  |  |  |
| Saturniidae | 30 | *Antheraea* + *Janiodes* | 100 | 100 | 98 | 88 | 83 |  |
|  | 31 | 29 + 30 |  |  |  |  |  |  |
| Anthelidae + Carthaeidae | 32 | *Chelepteryx* + *Carthaea* | 61 |  |  |  |  |  |
| Bombycidae 2 | 33 | *Phiditia* + *Prismosticta* | 52 | 75 |  |  |  |  |
|  | 34 | 32 + 33 | 88 | 70 |  |  |  |  |
|  | 35 | 31 + 34 |  |  |  |  |  |  |
| Eupterotidae + Brahmaeidae | 36 | *Apha* +*Acanthobrahmea* | 86 |  |  | 71 |  |  |
| Bombycidae 3 | 37 | *Apatelodes* + *Olceclostera* | 100 | 100 | 100 |  | 100 | 94 |
|  | 38 | 36 + 37 | 73 |  |  |  |  |  |
|  | 39 | 35 + 38 |  |  |  |  |  |  |
| Lasiocampidae | 40 | *Lasiocampa* + *Macrothylacia* | 50 |  | 59 | 52 |  |  |
|  | 40A | *Lasiocampa* + *Heteropacha* |  | 72 |  |  |  |  |
| Lasiocampidae | 41 | *Lasiocampa* + *Macrothylacia* + *Heteropacha* | 100 |  | 70 | 98 |  |  |
| Lasiocampidae | 42 | 41 + *Malacosoma* | 100 | 100 | 76 | 69 | 78 | 55 |
|  | 42A | 42 + *Poecilocampa* |  |  |  |  | 55 |  |
| Lasiocampidae | 43 | *Artace + Tolype* | 100 | 100 | 98 | 99 |  | 93 |
| Lasiocampidae | 43A | 42 + 43 |  |  | 69 |  |  |  |
| Lasiocampidae | 44 | 43 + *Poecilocampa* | 58 | 70 |  |  |  |  |
|  | 45 | 42 + 44 | 100 | 100 | 93 |  |  |  |
|  | 46 | 39 + 45 | 58 |  |  |  |  |  |
|  | 47 | 27 + 46 |  |  |  |  |  |  |
| Mimallonidae | 48 | *Druentia + Lacosoma* | 53 |  | 99 |  |  |  |
|  | 48A | *Trogoptera + Lacosoma* |  | 94 |  | 85 | 74 |  |
| Mimallonidae | 49 | *Druentia + Lacosoma* + *Trogoptera* | 100 | 98 | 94 | 99 | 97 | 99 |
| Cimeliidae | 50 | 49 + *Axia* |  |  |  |  |  |  |
| Drepanidae | 51 | *Pseudothyatira + Cyclidia* | 65 |  |  |  |  |  |
|  | 51A | *Pseudothyatira + Oreta* |  | 73 |  |  |  |  |
| Drepanidae | 52 | *Pseudothyatira + Cyclidia* + *Oreta* |  | 52 |  |  |  |  |
| Doidae | 53 | 52 + *Doa* |  |  |  |  |  |  |
|  | 54 | 50 + 53 |  |  |  |  |  |  |
|  | 55 | 47 + 54 |  |  |  |  |  |  |
| Crambidae | 56 | *Rupela* + *Scirpophaga* | 80 |  |  | 80 |  |  |
| Crambidae | 56A | *Rupela* + *Petrophila* |  | 72 |  |  |  |  |
| Crambidae | 57 | 56 + *Petrophila* | 80 |  |  |  |  |  |
| Crambidae | 58 | *Catoptria* + *Eudonia* | 100 | 62 | 57 | 55 | 58 |  |
| Crambidae | 59 | 57 + 58 | 100 |  |  |  |  |  |
| Crambidae | 59A | 56A + 58 (excluding *Scirpophaga*) |  | 70 |  |  |  |  |
| Crambidae | 60 | *Phaeodropsis* + *Mesocondyla* | 100 | 100 | 93 | 91 | 81 |  |
| Crambidae | 61 | 59 or 59A + 60 | 100 | 95 |  |  |  |  |
| Pyralidae | 62 | *Macrotheca* + *Monoloxis* | 91 |  |  | 52 |  |  |
| Pyralidae | 63 | *Accintapubes* + *Gauna* | 86 |  |  |  |  |  |
| Pyralidae | 64 | 63 + *Plodia* | 88 | 76 |  |  |  |  |
| Pyralidae | 64A | 64 + *Macrotheca* |  | 50 |  |  |  |  |
| Pyralidae | 65 | 62 + 64 | 99 | 91 |  |  |  |  |
| Pyraloidea | 66 | 61 + 65 | 65 | 73 |  |  |  |  |
|  | 67 | 55 + 66 |  |  |  |  |  |  |
| Limacodidae | 68 | *Apoda* + *Euclea* | 100 | 99 | 71 | 68 |  |  |
| Dalceridae | 69 | *Dalcerides* + *Acraga* | 100 | 100 | 99 | 99 |  | 98 |
|  | 70 | 68 + 69 |  |  |  |  |  |  |
| Megalopygidae + Aididae | 71 | *Lagoa* + *Aidos* | 100 | 85 |  |  |  |  |
| Lacturidae 1 | 72 | 71 + *Lactura* |  |  |  |  |  |  |
|  | 73 | 70 + 72 |  |  |  |  |  |  |
| Zygaenidae | 74 | *Eterusia* + *Pollanisus* | 65 | 52 |  |  |  | 57 |
| Zygaenidae | 75 | 74 + *Pryeria* | 71 |  |  |  |  |  |
| Lacturidae 2 | 76 | 75 + unidentified Lacturidae |  |  |  |  |  |  |
|  | 77 | 73 + 76 | 76 |  |  |  |  |  |
| Cyclotornidae + Epipyropidae | 78 | *Cyclotorna* + *Fulgoraecia* |  |  |  |  |  |  |
|  | 79 | 77 + 78 |  |  |  |  |  |  |
| Castniidae | 80 | *Amauta* + *Synemon* | 100 | 99 | 85 | 87 | 91 | 99 |
| Cossidae 1 | 81 | 80 + *Prionoxystus* | 60 |  |  |  |  |  |
|  | 82 | 79 + 81 |  |  |  |  |  |  |
| Sesiidae | 83 | *Melittia* + *Podosesia* | 100 |  | 67 | 54 | 80 |  |
| Cossidae 2 | 84 | 83 + *Zeuzera* |  |  |  |  |  |  |
|  | 85 | 82 + 84 |  |  |  |  |  |  |
| Nymphalidae | 86 | *Asterocampa* + *Phyciodes* | 100 | 99 | 55 | 76 |  | 59 |
| Pieridae | 87 | *Colias* + *Pieris* | 100 | 99 | 70 | 80 | 86 |  |
|  | 88 | 86 + 87 | 68 |  |  |  |  |  |
| Hesperiidae | 89 | *Astraptes* + *Urbanus* | 100 | 100 | 100 | 99 |  | 98 |
| Hedylidae | 90 | *Macrosoma* + *Macrosoma* | 100 | 100 |  | 100 | 100 | 91 |
|  | 91 | 89 + 90 | 68 |  |  |  |  |  |
|  | 92 | 88 + 91 | 50 |  |  |  |  |  |
| Thyrididae | 93 | *Dysodia* + *Pentina* |  |  |  |  |  |  |
| Thyrididae | 93A | *Dysodia* + *Rhodoneura* |  |  |  | 53 |  |  |
| Thyrididae | 94 | *Dysodia* + *Pentina* + *Rhodoneura* | 99 | 52 |  |  |  |  |
|  |  | *Dysodia* + *Rhodoneura* |  |  |  |  |  |  |
|  | 95 | 92 + 94 |  |  |  |  |  |  |
| Papilionidae | 96 | *Eurytides* + *Papilio* | 100 | 93 |  | 60 |  | 67 |
| Callidulidae | 97 | 96 + *Pterodecta* |  |  |  |  |  |  |
|  | 98 | 95 + 97 |  |  |  |  |  |  |
| Carposinidae + Hyblaeidae | 99 | *Sosineura* + *Hyblaea* |  |  |  |  |  |  |
|  | 100 | 98 + 99 |  |  |  |  |  |  |
|  | 101 | 85 + 100 |  |  |  |  |  |  |
|  | 102 | 67 + 101 |  |  |  |  |  |  |
| Cosmopterigidae + Elachistidae | 103 | *Euclemensia* + *Ethmia* |  |  |  |  |  |  |
| Pterophoridae | 104 | 103 + *Emmelina* |  |  |  |  |  |  |
|  | 105 | 102 + 104 |  |  |  |  |  |  |
| Tortricidae | 106 | *Argyrotaenia* + *Pandemis* | 100 | 91 | 53 | 82 | 87 | 79 |
| Tortricidae | 107 | 106 + *Clepsis* | 100 | 100 | 99 | 97 | 97 | 84 |
| Tortricidae | 107A | 107 + *Aethes* |  |  |  |  | 57 |  |
| Tortricidae | 108 | *Platynota* + *Anacrusis* | 70 |  | 65 |  | 56 |  |
| Tortricidae | 109 | 107 + 108 | 81 | 74 | 60 |  |  |  |
| Tortricidae | 110 | 109 + *Aethes* | 99 |  | 52 | 81 | 74 |  |
| Tortricidae | 111 | *Epiblemma* + *Pelochrista* | 100 | 100 | 94 | 100 | 100 | 100 |
| Tortricidae | 112 | 111 + *Cydia* | 100 | 100 | 97 | 89 | 100 | 59 |
| Tortricidae | 113 | 110 + 112 | 100 | 100 | 98 | 98 | 89 |  |
|  | 114A | 113 + *Syngria* |  |  |  | 51 |  |  |
|  | 114 | 105 + 113 |  |  |  |  |  |  |
| Yponomeutidae | 115 | *Atteva* + *Eucalantica* | 78 |  |  |  |  |  |
| Gracillariidae + Yponomeutidae | 116 | 115 + *Caloptilia* | 50 | 53 |  |  |  |  |
|  | 117 | 116 + 114 |  |  |  |  |  |  |
| Alucitidae + Urodidae | 118 | *Alucita* + *Urodus* |  |  |  |  |  |  |
|  | 119 | 117 + 118 |  |  |  |  |  |  |
| Choreutidae | 120 | 119 + *Hemerophila* |  |  |  |  |  |  |
| Tineidae | 121 | 120 *+Tineola* + *Tinea* | 100 | 100 | 100 | 98 | 100 | 99 |
|  | | |  |  |  |  |  |  |
|  | | | All 5 genes | CAD | DDC | Enolase | Period | Wingless |
| Number of nucleotides in data set | | | 6759 | 2928 | 1308 | 1134 | 987 | 402 |
| Total number of taxa yielding sequences | | | 123 | 123 | 120 | 113 | 112 | 117 |
| Total number of groupings with ≥50% BP | | | 82 | 58 | 30 | 35 | 26 | 20 |
| Total number of groupings in conflict with all-nt tree and supported by ≥50% BP | | | N/A | 6 | 3 | 3 | 2 | 1 |

**Figure A1**. (next page). This figure is identical to Fig. 3 in the main text, except that the nodes in the phylogram on the right have been numbered to correspond to the node numbers used in Table A1. The original legend is reprinted below.

## Best 123-taxon ML tree found for nt123

The ML nt123 topology is shown, with bootstrap values (BP) above branches (cladogram on left) separately calculated for ML nt123, ML nt12, and NoLRall2 + nt2, posterior probabilities from Bayesian nt123 analysis below branches. Dashes denote BP < 50%; brackets around BP or posterior probability mean group not recovered in the best ML tree for that partition and analysis. Branch lengths of the phylogram (right side) are proportional to total nucleotide change in ML nt123 tree. Major clade assignment (column to right of taxon names) according to working hypothesis (Fig. 2A): M = Macrolepidoptera; O-M = non-macrolepidopteran Obtectomera; A-O = non-obtectomeran Apoditrysia; D-A= non-apoditrysian Ditrysia.


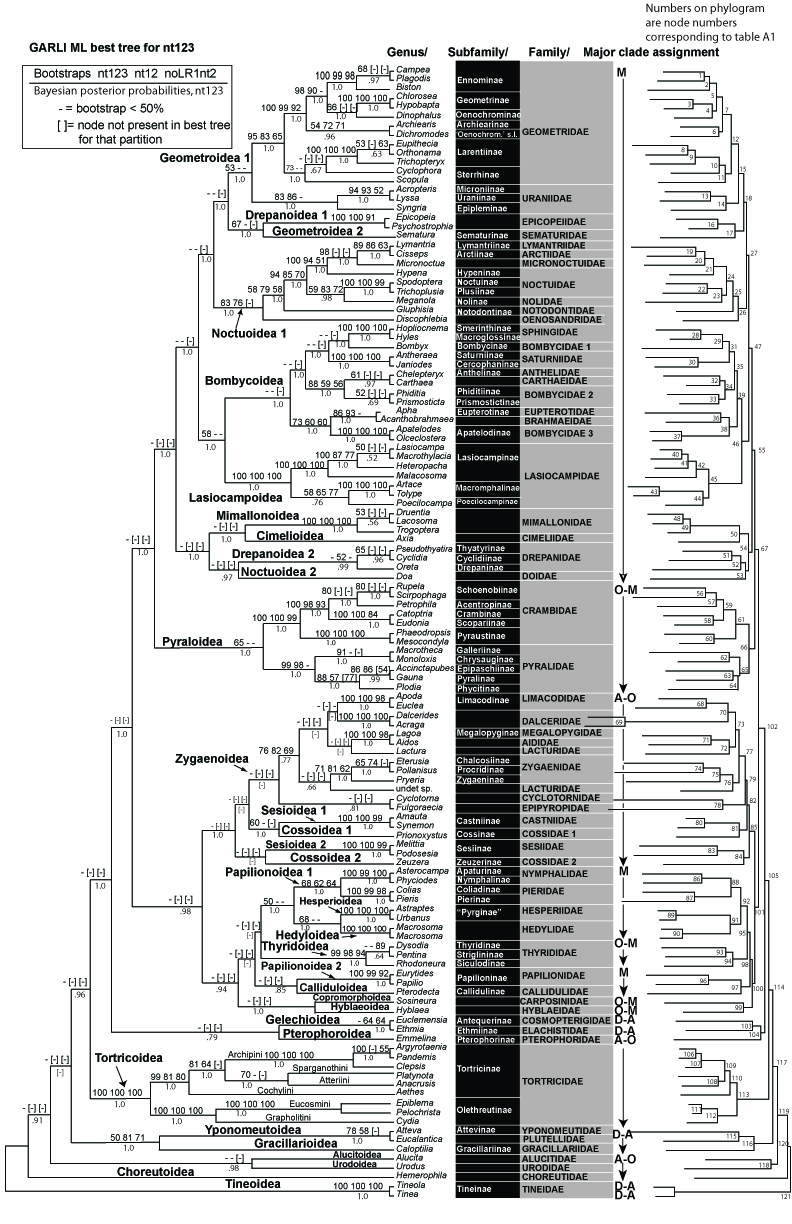


**Analysis and discussion**

Summed across the single-gene analyses, individual genes provided a total of 173 bootstrap values of ≥50%, with individual genes contributing in approximate proportion to their sequence length (Table A1). In just 15 of those 173 cases did the individual gene grouping conflict with the all-gene tree. For no node on the all-gene tree were there two or more genes supporting different alternatives to that grouping, each with ≥50% bootstrap. We found just two instances of individual gene groupings that conflicted strongly (bootstrap support of 70% or more) with other individual genes, with the all-gene result, or with conventional understanding of relationships. Overall, then, conflict among genes, especially strong conflict, appears to be rare in this data set. A similar conclusion was previously reached for these same genes in a partially overlapping taxon set (Regier et al. 2008).

It is worth examining the two cases of strong conflict more closely. One involves the three representatives of Mimallonidae (nodes 48, 49). CAD, enolase, and period favor pairing of *Trogoptera* and *Lacosoma* to the exclusion of *Druentia*, with bootstrap supports of 94, 85 and 74 respectively. DDC, in contrast, pairs *Lacosoma* with *Druentia*, with 99% bootstrap. The all-genes tree weakly favors the DDC solution, with 53% bootstrap. This looks like real conflict among gene trees, possibly attributable to lineage sorting, strong enough to prevent resolution of the species tree under gene concatenation.

The second case lies within Pyraloidea: Crambidae (nodes 56-59). As can be seen in the excerpt from Fig. 3 below, the five-gene tree groups the two representatives of Schoenobiinae, *Rupela* and *Scirpophaga*, and these within two additional nodes subtending three other subfamilies, all with 80-100% bootstrap support. The *Rupela*/ *Scirpophaga* pairing is also strongly supported by enolase alone (BP=80%). CAD alone, in contrast (see Fig. A2 below), places *Scirpophaga* several nodes down, as the sister group of a lineage supported by 70% bootstrap. In this case, the all-gene tree seems more likely to be correct, as morphological synapomorphies strongly support the monophyly of Schoenobiinae (Solis 2007).

**Figure A2**, showing the conflicting placement of *Scirpophaga* by CAD analyzed alone.


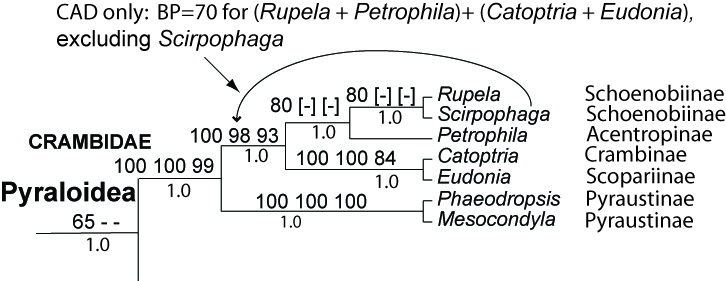


References:

Regier, J.C., Cook, C.P., Mitter, C. & Hussey, A. 2008. A phylogenetic study of the ‘‘bombycoid complex’’ (Lepidoptera) using five protein-coding nuclear genes, with comments on the problem of macrolepidopteran phylogeny. Systematic Entomology 33:175–189.

Solis, M. A. 2007. Phylogenetic studies and modern classification of the Pyraloidea (Lepidoptera). Revista Colombiana de Entomología 33: 1-9
