# Supplementary material for: Toward reconstructing the evolution of advanced moths and butterflies (Lepidoptera: Ditrysia): an initial molecular study
Source: BMC Evol Biol. 2009 Dec 2;9:280. doi: 10.1186/1471-2148-9-280 (PMC2796670; doi:10.1186/1471-2148-9-280)
Supplement: Additional file 5 — Nt123 Bayesian analysis, partitioned noLRall2 + nt2 vs. LR + nt3. Majority rule consensus of trees sampled from partitioned Bayesian analysis of nt123 with partitions noLRall2 + nt2 versus LRall2 + nt3. Two runs, 10106 trees sampled from each, with standard deviation of split frequencies < 0.01. [file 1471-2148-9-280-S5.PDF]

Additional File 5. Nt123 Bayesian analysis, partitioned noLRall2 + nt2 vs. LR + nt3, majority rule consensus tree from two samples, 10106 trees each, from two samples, taken after these had converged, as indicated by standard deviation of split frequencies less than 0.01.

```
                               /---- BOMBYCOIDEA_Eupterotidae_Apha_aequalis_AAEQ
                            /100
                            |  \---- BOMBYCOIDEA_Brahmaeidae_Acanthobrahmaea_europaea_AEURO
                 /--------100
                 |          |  /---- BOMBYCOIDEA_Bombycidae_Apatelodes_torrefacta_ATOR
                 |          \100
                 |             \---- BOMBYCOIDEA_Bombycidae_Olceclostera_seraphica_OSERA
                 |
                 |             /---- BOMBYCOIDEA_Saturniidae_Antheraea_polyphemus_APOL2
                 |      /----100
              /100      |      \---- BOMBYCOIDEA_Saturniidae_Janiodes_sp_JCER
              |  |      |
              |  |   /100   /------- BOMBYCOIDEA_Bombycidae_Bombyx_mori_BMOR1
              |  |   |  |   |
              |  |   |  \-100  /---- BOMBYCOIDEA_Sphingidae_Hopliocnema_brachycera_HBR
              |  |   |      \100
              |  |   |         \---- BOMBYCOIDEA_Sphingidae_Hyles_lineata_HLIN
              |  \-100
              |      |         /---- LASIOCAMPOIDEA_Anthelidae_Chelepteryx_collesi_CCOL
              |      |      /-97
              |      |      |  \---- BOMBYCOIDEA_Carthaeidae_Carthaea_saturnioides_TB032177
              |      \----100
   /--------100             |  /---- BOMBYCOIDEA_Bombycidae_Phiditia_sp_PBO
   |          |             \-69
   |          |                \---- BOMBYCOIDEA_Bombycidae_Prismosticta_fenestrata_PFENE
   |          |
   |          |                /---- LASIOCAMPOIDEA_Lasiocampidae_Artace_cribraria_ACRI
   |          |             /100
   |          |             |  \---- LASIOCAMPOIDEA_Lasiocampidae_Tolype_notialis_TNOT
   |          |      /-----76
   |          |      |      \------- LASIOCAMPOIDEA_Lasiocampidae_Poecilocampa_populi_PPOP
   |          |      |
   |          |      |      /------- LASIOCAMPOIDEA_Lasiocampidae_Heteropacha_rileyana_HRIL
   |          \----100      |
   |                 |  /-100  /---- LASIOCAMPOIDEA_Lasiocampidae_Lasiocampa_quercus_LQUERCUS
   |                 |  |   \-52
   |                 \100      \---- LASIOCAMPOIDEA_Lasiocampidae_Macrothylacia_rubi_MRUBI
   |                    |
   |                    \----------- LASIOCAMPOIDEA_Lasiocampidae_Malacosoma_americanum_MAME2
   |
   |                           /---- GEOMETROIDEA_Geometridae_Archiearis_parthenias_APAR
   |                 /--------96
   |                 |         \---- GEOMETROIDEA_Geometridae_Dichromodes_sp_DICH
   |                 |
   |                 |      /------- GEOMETROIDEA_Geometridae_Biston_betularia_BBET
   |                 |      |
   |             /-100  /-100  /---- GEOMETROIDEA_Geometridae_Campaea_perlata_CPER
   |             |   |  |   \-97
   |             |   |  |      \---- GEOMETROIDEA_Geometridae_Plagodis_fervidaria_PFER
   |             |   |  |
   |             |   \100      /---- GEOMETROIDEA_Geometridae_Chlorosea_margaretaria_CMAR
/100             |      |   /100
|  |             |      |   |  \---- GEOMETROIDEA_Geometridae_Hypobapta_xenomorpha_HXEN
|  |             |      \-100
|  |          /100          \------- GEOMETROIDEA_Geometridae_Dinophalus_lechriomita_DLEC
|  |          |  |
|  |          |  |      /----------- GEOMETROIDEA_Geometridae_Cyclophora_sp_CYTR
|  |          |  |      |
|  |          |  |      |      /---- GEOMETROIDEA_Geometridae_Eupithecia_acutipennis_EUAC
|  |          |  |   /-67   /-96
|  |          |  |   |  |   |  \---- GEOMETROIDEA_Geometridae_Orthonama_obstipata_ONST
|  |      /-100  |   |  \-100
|  |      |   |  \-100      \------- GEOMETROIDEA_Geometridae_Trichopteryx_carpinata_TRCA
|  |      |   |      |
|  |      |   |      \-------------- GEOMETROIDEA_Geometridae_Scopula_limboundata_SCLI
|  |      |   |
|  |      |   |             /------- GEOMETROIDEA_Uraniidae_Syngria_druidaria_SDRU
```

```
                              |  |      |   |             |
                              |  |   /100   \-----------100  /---- GEOMETROIDEA_Uraniidae_Acropteris_sparsaria_ACRO
                              |  |   |  |                 \100
                              |  |   |  |                    \---- GEOMETROIDEA_Uraniidae_Lyssa_zampa_LZA
                              |  |   |  |
                              |  |   |  |                 /------- GEOMETROIDEA_Sematuridae_Sematura_luna_NOTHSP
                              |  |   |  |                 |
                              |  |   |  \---------------100  /---- DREPANOIDEA_Epicopeiidae_Epicopeia_hainesii_EHAI
                              |  |   |                    \100
                              |  |   |                       \---- DREPANOIDEA_Epicopeiidae_Psychostrophia_melangaria_PMEL
                          /-100  |   |
                          |   |  |   |                       /---- NOCTUOIDEA_Lymantriidae_Lymantria_dispar_LDI
                          |   |  |   |                    /100
                          |   |  \-100                |  \---- NOCTUOIDEA_Arctiidae_Cisseps_fulvicollis_CFU2
                          |   |      |                /-100
                          |   |      |                |   \------- NOCTUOIDEA_Micronoctuidae_Micronoctua_karsholti_MKA
                          |   |      |             /100
                          |   |      |             |  \----------- NOCTUOIDEA_Noctuidae_Hypena_scabra_PSC2
                          |   |      |             |
                          |   |      |         /-100         /---- NOCTUOIDEA_Noctuidae_Spodoptera_frugiperda_SFR
                          |   |      |         |   |      /100
                          |   |      |         |   |      |  \---- NOCTUOIDEA_Noctuidae_Trichoplusia_ni_TNI
                          |   |      |      /100   \-----98
                          |   |      |      |  |          \------- NOCTUOIDEA_Nolidae_Meganola_phylla_MMI1
                          |   |      |      |  |
                          |   |      \----100  \------------------ NOCTUOIDEA_Notodontidae_Gluphisia_septentrionis_GSEP
                          |   |             |
                          |   |             \--------------------- NOCTUOIDEA_Oenosandridae_Discophlebia_sp_DISC
                          |   |
                          |   |                       /----------- CIMELIOIDEA_Cimeliidae_Axia_margarita_AXSP
                          |   |                       |
                          |   |                    /100   /------- MIMALLONOIDEA_Mimallonidae_Druentia_alsa_DALS
                          |   |                    |  |   |
                       /100   |                    |  \-100  /---- MIMALLONOIDEA_Mimallonidae_Lacosoma_chiridota_LCH2
                       |  |   |                    |      \-56
                       |  |   |                    |         \---- MIMALLONOIDEA_Mimallonidae_Trogoptera_salvita_TSAV
                       |  |   \------------------100
                       |  |                        |      /------- DREPANOIDEA_Drepanidae_Oreta_rosea_OROS
                       |  |                        |      |
                       |  |                        |  /--99  /---- DREPANOIDEA_Drepanidae_Pseudothyatira_cymatophoroides_PCYM
                       |  |                        |  |   \-96
                       |  |                        \-97      \---- DREPANOIDEA_Drepanidae_Cyclidia_substigmaria_modesta_CYSU
                       |  |                           |
                       |  |                           \----------- NOCTUOIDEA_Doidae_Doa_sp_DOA
                       |  |
                       |  |                               /------- PYRALOIDEA_Pyralidae_Plodia_interpunctella_PIN
                       |  |                               |
                       |  |                           /-100  /---- PYRALOIDEA_Pyralidae_Accinctapubes_albifasciata_ACAL
                       |  |                           |   \-99
                       |  |                           |      \---- PYRALOIDEA_Pyralidae_Gauna_aegusalis_GAEG
                       |  |                    /----100
                       |  |                    |      |      /---- PYRALOIDEA_Pyralidae_Macrotheca_sp_MACRO
                       |  |                    |      \----100
                       |  |                    |             \---- PYRALOIDEA_Pyralidae_Monoloxis_flavicintalis_MFLA
                       |  |                    |
                       |  \------------------100             /---- PYRALOIDEA_Crambidae_Phaeodropsis_alitemeralis_PPY122
                       |                       |   /-------100
                       |                       |   |         \---- PYRALOIDEA_Crambidae_Mesocondyla_dardusalis_PPY224
                       |                       |   |
                       |                       |   |         /---- PYRALOIDEA_Crambidae_Rupela_albina_PSH29
                       |                       \-100      /100
                       |                           |      |  \---- PYRALOIDEA_Crambidae_Scirpophaga_incertulas_SIN
                       |                           |  /-100
                       |                           |  |   \------- PYRALOIDEA_Crambidae_Petrophila_confusalis_PCON
                       |                           \100
                       |                              |      /---- PYRALOIDEA_Crambidae_Catoptria_oregonica_CAOR
                       |                              \----100
                       |                                     \---- PYRALOIDEA_Crambidae_Eudonia_spenceri_SCSP
                       |
                       |                                     /---- PAPILIONOIDEA_Nymphalidae_Asterocampa_celtis_ACLY
                       |                                  /100
                       |                                  |  \---- PAPILIONOIDEA_Nymphalidae_Phyciodes_tharos_PTHA
                   /-100                              /-100
```

```
       |   |                              |   |  /---- PAPILIONOIDEA_Pieridae_Colias_eurytheme_CEUR
       |   |                              |   \100
       |   |                              |      \---- PAPILIONOIDEA_Pieridae_Pieris_rapae_PRAP
       |   |                           /100
       |   |                           |  |      /---- HESPERIOIDEA_Hesperiidae_Astraptes_enotrus_AENO
       |   |                           |  |   /100
       |   |                           |  |   |  \---- HESPERIOIDEA_Hesperiidae_Urbanus_doryssus_UDO
       |   |                           |  \-100
       |   |                           |      |  /---- HEDYLOIDEA_Hedylidae_Macrosoma_conifera_MCON
       |   |                           |      \100
       |   |                           |         \---- HEDYLOIDEA_Hedylidae_Macrosoma_satellitiata_satellitiata_MSSA
       |   |                       /--88
       |   |                       |   |         /---- PAPILIONOIDEA_Papilionidae_Mimoides_branchus_MIBR
       |   |                       |   |      /100
       |   |                       |   |      |  \---- PAPILIONOIDEA_Papilionidae_Papilio_glaucus_PGLA
       |   |                       |   |  /--85
       |   |                       |   |  |   \------- CALLIDULOIDEA_Callidulidae_Pterodecta_felderi_PTFE
       |   |                       |   |  |
       |   |                /-----94   \-65      /---- THYRIDOIDEA_Thyrididae_Dysodia_sp_DYSO
       |   |                |      |      |   /-64
       |   |                |      |      |   |  \---- THYRIDOIDEA_Thyrididae_Pentina_flammans_PFLA
       |   |                |      |      \-100
       |   |                |      |          \------- THYRIDOIDEA_Thyrididae_Rhodoneura_terminalis_LTE
       |   |                |      |
       |   |                |      |             /---- COPROMORPHOIDEA_Carposinidae_Sosineura_mimica_SMIM
       |   |                |      \-----------100
       |   |                |                    \---- HYBLAEOIDEA_Hyblaeidae_Hyblaea_firmamentum_HYFM
       |   |                |
       |   |                |                    /---- ZYGAENOIDEA_Limacodidae_Apoda_biguttata_ABIG2
       |   |                |          /-------100
       |   |                |          |         \---- ZYGAENOIDEA_Limacodidae_Euclea_delphinii_EDEL3
       |   |                |          |
       |   |                |          |         /---- ZYGAENOIDEA_Dalceridae_Dalcerides_ingenita_DING2
       |   |                |          +-------100
    /100   |                |          |         \---- ZYGAENOIDEA_Dalceridae_Acraga_philetera_DSP
    |  |   |                |          |
    |  |   |                |          |         /---- ZYGAENOIDEA_Zygaenidae_Eterusia_aedea_ETSP
    |  |   \---------------98          |      /100
    |  |                    |          |      |  \---- ZYGAENOIDEA_Zygaenidae_Pollanisus_sp_POSP
    |  |                    |      /--77  /-100
    |  |                    |      |   |  |   \------- ZYGAENOIDEA_Zygaenidae_Pryeria_sinica_PSIN
    |  |                    |      |   +-66
    |  |                    |      |   |  \----------- ZYGAENOIDEA_Lacturidae_Gen_sp_LACT
    |  |                    |      |   |
    |  |                    |      |   |         /---- ZYGAENOIDEA_Megalopygidae_Lagoa_crispata_LCR2
    |  |                    |      |   +-------100
    |  |                    |   /-75   |         \---- ZYGAENOIDEA_Aididae_Aidos_osorius_AIOS
    |  |                    |   |  |   |
    |  |                    |   |  |   \-------------- ZYGAENOIDEA_Lacturidae_Lactura_subfervens_LSUB
    |  |                    |   |  |
    |  |                    |   |  |             /---- SESIOIDEA_Castniidae_Amauta_cacica_AMCA
    |  |                    |   |  |          /100
    |  |                    |   |  |          |  \---- SESIOIDEA_Castniidae_Synemon_plana_SPLA
    |  |                    |   |  \--------100
    |  |                    |   |             \------- COSSOIDEA_Cossidae_Prionoxystus_robiniae_PROB2
    |  |                    \-100
    |  |                        |                /---- ZYGAENOIDEA_Cyclotornidae_Cyclotorna_sp_CYSP
    |  |                        |             /-81
    |  |                        |             |  \---- ZYGAENOIDEA_Epipyropidae_Fulgoraecia_exigua_ESP2
    |  |                        +------------68
    |  |                        |             \------- COSSOIDEA_Cossidae_Zeuzera_coffeae_ZCOF
/--96  |                        |
|   |  |                        |                /---- SESIOIDEA_Sesiidae_Melittia_cucurbitae_MCUC2
|   |  |                        \--------------100
|   |  |                                         \---- SESIOIDEA_Sesiidae_Podosesia_syringae_PSY2
|   |  |
|   |  |                                         /---- GELECHIOIDEA_Cosmopterigidae_Euclemensia_bassettella_COLE
|   |  |                                      /100
|   |  |                                      |  \---- GELECHIOIDEA_Elachistidae_Ethmia_eupostica_EEU
|   |  \------------------------------------79
|   |                                         \------- PTEROPHOROIDEA_Pterophoridae_Emmelina_monodactyla_EMON
|   |
|   |                                         /------- TORTRICOIDEA_Tortricidae_Cydia_pomonella_CPO
```

```
          |   |                                         |
          |   |                              /--------100  /---- TORTRICOIDEA_Tortricidae_Epiblema_abruptana_BASP
          |   |                              |          \100
          |   |                              |             \---- TORTRICOIDEA_Tortricidae_Pelochrista_zomonana_EUSP
          |   |                              |
          |   |                              |             /---- TORTRICOIDEA_Tortricidae_Argyrotaenia_alisellana_ARGA
       /-91   |                              |          /100
       |  |   |                           /100          |  \---- TORTRICOIDEA_Tortricidae_Pandemis_limitata_CNSP
       |  |   |                           |  |      /-100
       |  |   |                           |  |      |   \------- TORTRICOIDEA_Tortricidae_Clepsis_melalucana_CRS
       |  |   |                           |  |   /100
       |  |   |                           |  |   |  |      /---- TORTRICOIDEA_Tortricidae_Platynota_idaeusalis_PIDA2
       |  |   |                           |  |   |  \----100
       |  |   |                           |  \-100         \---- TORTRICOIDEA_Tortricidae_Anacrusis_nephrodes_ANNE
       |  |   \--------------------------89      |
       |  |                               |      \--------------- TORTRICOIDEA_Tortricidae_Aethes_promptana_AESP
       |  |                               |
   /-100  |                               |                /---- YPONOMEUTOIDEA_Yponomeutidae_Atteva_punctella_ATPU2
   |   |  |                               |             /100
   |   |  |                               |             |  \---- YPONOMEUTOIDEA_Plutellidae_Eucalantica_sp_YSP
   |   |  |                               \-----------100
   |   |  |                                             \------- GRACILLARIOIDEA_Gracillariidae_Caloptilia_bimaculatella_CBIM
   |   |  |
   |   |  |                                                /---- ALUCITOIDEA_Alucitidae_Alucita_sp_ALSP
---+   |  \-----------------------------------------------98
   |   |                                                   \---- URODOIDEA_Urodidae_Urodus_decens_URSP
   |   |
   |   \-------------------------------------------------------- CHOREUTOIDEA_Choreutidae_Hemerophila_felis_HFEL
   |
   +------------------------------------------------------------ TINEOIDEA_Tineidae_Tineola_bisselliella_TBI3
   |
   \------------------------------------------------------------ TINEOIDEA_Tineidae_Tinea_columbariella_TCO2
```
