# Supplementary material for: Toward reconstructing the evolution of advanced moths and butterflies (Lepidoptera: Ditrysia): an initial molecular study
Source: BMC Evol Biol. 2009 Dec 2;9:280. doi: 10.1186/1471-2148-9-280 (PMC2796670; doi:10.1186/1471-2148-9-280)
Supplement: Additional file 7 — 123-taxon ML tree & bootstrap consensus tree for nt12. Part A: nt12 best ML tree found in 10,000 replicate GARLI searches, GTR + G + I model, phylogram format. Part B: nt12 bootstrap majority rule consensus tree, generated in PAUP, from 1000 GARLI ML bootstrap replicates, GTR + G + I model. [file 1471-2148-9-280-S7.PDF]

Additional File 7, Part A. Nt12 best ML tree found in 10,000 replicate GARLI runs,GTR +G+I model.

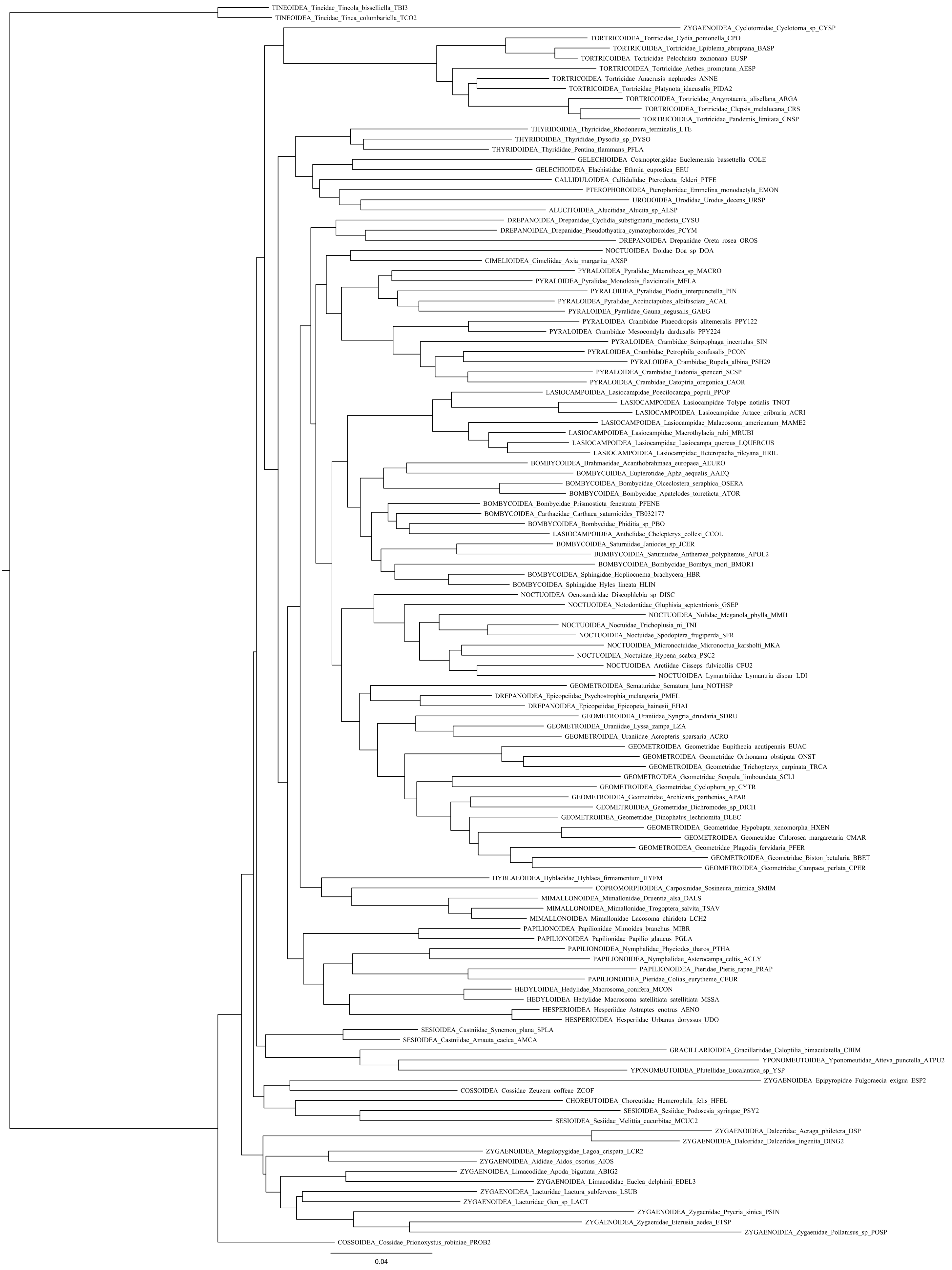

Additional File 7, Part B. nt12 bootstrap majority rule consensus tree (LE option on), generated in PAUP, from 1000 GARLI ML bootstrap replicates, GTR+G+I model. Bootstrap values are embedded in branches.

```
                                                         /------ BOMBYCOIDEA_Saturniidae_Antheraea_polyphemus_APOL2
                                                  /-100--+------ BOMBYCOIDEA_Saturniidae_Janiodes_sp_JCER
                                      /----28-----+      /------ BOMBYCOIDEA_Sphingidae_Hopliocnema_brachycera_HBR
                                      |           \-100--+------ BOMBYCOIDEA_Sphingidae_Hyles_lineata_HLIN
                                      |                  /------ LASIOCAMPOIDEA_Anthelidae_Chelepteryx_collesi_CCOL
                               /--21--+           /--59--+------ BOMBYCOIDEA_Bombycidae_Phiditia_sp_PBO
                               |      |     /-48--+------------- BOMBYCOIDEA_Carthaeidae_Carthaea_saturnioides_TB032177
                         /-36--+      \-60--+------------------- BOMBYCOIDEA_Bombycidae_Prismosticta_fenestrata_PFENE
                         |     \-------------------------------- BOMBYCOIDEA_Bombycidae_Bombyx_mori_BMOR1
                   /-30--+                               /------ BOMBYCOIDEA_Eupterotidae_Apha_aequalis_AAEQ
                   |     |                        /--93--+------ BOMBYCOIDEA_Brahmaeidae_Acanthobrahmaea_europaea_AEURO
                   |     \-----------59-----------+      /------ BOMBYCOIDEA_Bombycidae_Apatelodes_torrefacta_ATOR
                   |                              \-100--+------ BOMBYCOIDEA_Bombycidae_Olceclostera_seraphica_OSERA
                   |                                     /------ LASIOCAMPOIDEA_Lasiocampidae_Heteropacha_rileyana_HRIL
             /-42--+                              /--94--+------ LASIOCAMPOIDEA_Lasiocampidae_Lasiocampa_quercus_LQUERCUS
             |     |                        /-87--+------------- LASIOCAMPOIDEA_Lasiocampidae_Macrothylacia_rubi_MRUBI
             |     |                  /-100-+------------------- LASIOCAMPOIDEA_Lasiocampidae_Malacosoma_americanum_MAME2
             |     |                  |                  /------ LASIOCAMPOIDEA_Lasiocampidae_Artace_cribraria_ACRI
             |     \-------100--------+           /-100--+------ LASIOCAMPOIDEA_Lasiocampidae_Tolype_notialis_TNOT
             |                        \----66-----+------------- LASIOCAMPOIDEA_Lasiocampidae_Poecilocampa_populi_PPOP
             |                                           /------ NOCTUOIDEA_Lymantriidae_Lymantria_dispar_LDI
      /--13--+                                    /--84--+------ NOCTUOIDEA_Arctiidae_Cisseps_fulvicollis_CFU2
      |      |                              /-94--+      /------ NOCTUOIDEA_Micronoctuidae_Micronoctua_karsholti_MKA
      |      |                              |     \--45--+------ NOCTUOIDEA_Noctuidae_Hypena_scabra_PSC2
      |      |                        /-83--+            /------ NOCTUOIDEA_Noctuidae_Spodoptera_frugiperda_SFR
      |      |                        |     |     \-100--+------ NOCTUOIDEA_Noctuidae_Trichoplusia_ni_TNI
      |      |                 /--75--+     \-80--+------------- NOCTUOIDEA_Nolidae_Meganola_phylla_MMI1
      |      \-------74--------+      \------------------------- NOCTUOIDEA_Notodontidae_Gluphisia_septentrionis_GSEP
      |                        \-------------------------------- NOCTUOIDEA_Oenosandridae_Discophlebia_sp_DISC
      |                                                  /------ GEOMETROIDEA_Geometridae_Biston_betularia_BBET
      |                                           /--66--+------ GEOMETROIDEA_Geometridae_Campaea_perlata_CPER
      |                                     /-100-+------------- GEOMETROIDEA_Geometridae_Plagodis_fervidaria_PFER
      |                               /-51--+            /------ GEOMETROIDEA_Geometridae_Chlorosea_margaretaria_CMAR
/-14--+                        /--90--+     \----100-----+------ GEOMETROIDEA_Geometridae_Hypobapta_xenomorpha_HXEN
|     |                        |      \------------------------- GEOMETROIDEA_Geometridae_Dinophalus_lechriomita_DLEC
|     |                  /-100-+                         /------ GEOMETROIDEA_Geometridae_Archiearis_parthenias_APAR
|     |                  |     \-----------74------------+------ GEOMETROIDEA_Geometridae_Dichromodes_sp_DICH
|     |                  |                               /------ GEOMETROIDEA_Geometridae_Orthonama_obstipata_ONST
|     |            /-84--+                        /--70--+------ GEOMETROIDEA_Geometridae_Trichopteryx_carpinata_TRCA
|     |            |     |                  /-100-+------------- GEOMETROIDEA_Geometridae_Eupithecia_acutipennis_EUAC
|     |            |     \--------41--------+            /------ GEOMETROIDEA_Geometridae_Cyclophora_sp_CYTR
|     |      /-35--+                        \-----75-----+------ GEOMETROIDEA_Geometridae_Scopula_limboundata_SCLI
|     |      |     |                                     /------ GEOMETROIDEA_Uraniidae_Acropteris_sparsaria_ACRO
|     |      |     |                              /--92--+------ GEOMETROIDEA_Uraniidae_Lyssa_zampa_LZA
|     \--47--+     \--------------86--------------+------------- GEOMETROIDEA_Uraniidae_Syngria_druidaria_SDRU
```

```
                                                  |            |                                           /------ DREPANOIDEA_Epicopeiidae_Epicopeia_hainesii_EHAI
                                            /--1--+            |                                    /-100--+------ DREPANOIDEA_Epicopeiidae_Psychostrophia_melangaria_PMEL
                                            |     |            \------------------30----------------+------------- GEOMETROIDEA_Sematuridae_Sematura_luna_NOTHSP
                                            |     |                                                        /------ PYRALOIDEA_Crambidae_Rupela_albina_PSH29
                                            |     |                                                 /--61--+------ PYRALOIDEA_Crambidae_Petrophila_confusalis_PCON
                                            |     |                                           /-44--+      /------ PYRALOIDEA_Crambidae_Catoptria_oregonica_CAOR
                                            |     |                                     /-98--+     \-100--+------ PYRALOIDEA_Crambidae_Eudonia_spenceri_SCSP
                                            |     |                                     |     \------------------- PYRALOIDEA_Crambidae_Scirpophaga_incertulas_SIN
                                            |     |                              /-100--+                  /------ PYRALOIDEA_Crambidae_Phaeodropsis_alitemeralis_PPY122
                                            |     |                              |      \-------100--------+------ PYRALOIDEA_Crambidae_Mesocondyla_dardusalis_PPY224
                                     /--5---+     |                              |                         /------ PYRALOIDEA_Pyralidae_Accinctapubes_albifasciata_ACAL
                                     |      |     |                        /-36--+                  /--89--+------ PYRALOIDEA_Pyralidae_Gauna_aegusalis_GAEG
                                     |      |     |                        |     |            /-58--+------------- PYRALOIDEA_Pyralidae_Plodia_interpunctella_PIN
                                     |      |     |                        |     \-----98-----+            /------ PYRALOIDEA_Pyralidae_Macrotheca_sp_MACRO
                                     |      |     \------------4-----------+                  \-----36-----+------ PYRALOIDEA_Pyralidae_Monoloxis_flavicintalis_MFLA
                                     |      |                              |                               /------ CIMELIOIDEA_Cimeliidae_Axia_margarita_AXSP
                               /-25--+      |                              \---------------24--------------+------ NOCTUOIDEA_Doidae_Doa_sp_DOA
                               |     |      |                                                              /------ DREPANOIDEA_Drepanidae_Oreta_rosea_OROS
                               |     |      |                                                       /--52--+------ DREPANOIDEA_Drepanidae_Pseudothyatira_cymatophoroides_PCYM
                               |     |      \---------------------------44--------------------------+------------- DREPANOIDEA_Drepanidae_Cyclidia_substigmaria_modesta_CYSU
                         /-19--+     |                                                                     /------ MIMALLONOIDEA_Mimallonidae_Lacosoma_chiridota_LCH2
                         |     |     |                                                              /--49--+------ MIMALLONOIDEA_Mimallonidae_Trogoptera_salvita_TSAV
                         |     |     \------------------------------100-----------------------------+------------- MIMALLONOIDEA_Mimallonidae_Druentia_alsa_DALS
                         |     |                                                                           /------ COPROMORPHOIDEA_Carposinidae_Sosineura_mimica_SMIM
                         |     \------------------------------------31-------------------------------------+------ HYBLAEOIDEA_Hyblaeidae_Hyblaea_firmamentum_HYFM
                         |                                                                                 /------ PAPILIONOIDEA_Nymphalidae_Asterocampa_celtis_ACLY
                  /--4---+                                                                          /--97--+------ PAPILIONOIDEA_Nymphalidae_Phyciodes_tharos_PTHA
                  |      |                                                                    /-62--+      /------ PAPILIONOIDEA_Pieridae_Colias_eurytheme_CEUR
                  |      |                                                                    |     \--99--+------ PAPILIONOIDEA_Pieridae_Pieris_rapae_PRAP
                  |      |                                                              /-45--+            /------ HESPERIOIDEA_Hesperiidae_Astraptes_enotrus_AENO
                  |      |                                                              |     |     /-100--+------ HESPERIOIDEA_Hesperiidae_Urbanus_doryssus_UDO
                  |      |                                                              |     \-46--+      /------ HEDYLOIDEA_Hedylidae_Macrosoma_conifera_MCON
                  |      \------------------------------19------------------------------+           \-100--+------ HEDYLOIDEA_Hedylidae_Macrosoma_satellitiata_satellitiata_MSSA
            /-21--+                                                                     |                  /------ PAPILIONOIDEA_Papilionidae_Mimoides_branchus_MIBR
            |     |                                                                     \--------99--------+------ PAPILIONOIDEA_Papilionidae_Papilio_glaucus_PGLA
            |     |                                                                                        /------ ALUCITOIDEA_Alucitidae_Alucita_sp_ALSP
            |     |                                                                                 /--16--+------ PTEROPHOROIDEA_Pterophoridae_Emmelina_monodactyla_EMON
            |     |                                                                           /-15--+------------- CALLIDULOIDEA_Callidulidae_Pterodecta_felderi_PTFE
      /--4--+     |                                                                     /--8--+            /------ GELECHIOIDEA_Cosmopterigidae_Euclemensia_bassettella_COLE
      |     |     |                                                                     |     \-----68-----+------ GELECHIOIDEA_Elachistidae_Ethmia_eupostica_EEU
      |     |     \-----------------------------------6---------------------------------+                  /------ THYRIDOIDEA_Thyrididae_Dysodia_sp_DYSO
      |     |                                                                           |           /--46--+------ THYRIDOIDEA_Thyrididae_Pentina_flammans_PFLA
      |     |                                                                           \----98-----+------------- THYRIDOIDEA_Thyrididae_Rhodoneura_terminalis_LTE
      |     \----------------------------------------------------------------------------------------------------- URODOIDEA_Urodidae_Urodus_decens_URSP
      |                                                                                                    /------ TORTRICOIDEA_Tortricidae_Pandemis_limitata_CNSP
/--2--+                                                                                             /--55--+------ TORTRICOIDEA_Tortricidae_Clepsis_melalucana_CRS
|     |                                                                                       /-100-+------------- TORTRICOIDEA_Tortricidae_Argyrotaenia_alisellana_ARGA
|     |                                                                                 /-60--+            /------ TORTRICOIDEA_Tortricidae_Platynota_idaeusalis_PIDA2
```

```
             |     |                                                                          /--81--+     \-----39-----+------ TORTRICOIDEA_Tortricidae_Anacrusis_nephrodes_ANNE
             |     |                                                                          |      \------------------------- TORTRICOIDEA_Tortricidae_Aethes_promptana_AESP
             |     |                                                                    /-100-+                         /------ TORTRICOIDEA_Tortricidae_Epiblema_abruptana_BASP
             |     |                                                                    |     |                  /-100--+------ TORTRICOIDEA_Tortricidae_Pelochrista_zomonana_EUSP
             |     \---------------------------------20---------------------------------+     \-------100--------+------------- TORTRICOIDEA_Tortricidae_Cydia_pomonella_CPO
             |                                                                          \-------------------------------------- ZYGAENOIDEA_Cyclotornidae_Cyclotorna_sp_CYSP
             |                                                                                                          /------ ZYGAENOIDEA_Zygaenidae_Eterusia_aedea_ETSP
             |                                                                                                   /--69--+------ ZYGAENOIDEA_Zygaenidae_Pollanisus_sp_POSP
             |                                                                                             /-80--+------------- ZYGAENOIDEA_Zygaenidae_Pryeria_sinica_PSIN
             |                                                                                       /-45--+            /------ ZYGAENOIDEA_Lacturidae_Gen_sp_LACT
      /--18--+                                                                                       |     \-----39-----+------ ZYGAENOIDEA_Lacturidae_Lactura_subfervens_LSUB
      |      |                                                                                /--12--+                  /------ ZYGAENOIDEA_Megalopygidae_Lagoa_crispata_LCR2
      |      |                                                                                |      \-------100--------+------ ZYGAENOIDEA_Aididae_Aidos_osorius_AIOS
      |      |                                                                          /-82--+                         /------ ZYGAENOIDEA_Limacodidae_Apoda_biguttata_ABIG2
      |      |                                                                          |     |                  /-100--+------ ZYGAENOIDEA_Limacodidae_Euclea_delphinii_EDEL3
      |      |                                                                          |     \--------28--------+      /------ ZYGAENOIDEA_Dalceridae_Dalcerides_ingenita_DING2
      |      |                                                                    /--7--+                        \-100--+------ ZYGAENOIDEA_Dalceridae_Acraga_philetera_DSP
      |      |                                                                    |     |                               /------ SESIOIDEA_Sesiidae_Melittia_cucurbitae_MCUC2
      |      |                                                                    |     |                        /-100--+------ SESIOIDEA_Sesiidae_Podosesia_syringae_PSY2
/-----+      |                                                              /--1--+     \----------59------------+------------- CHOREUTOIDEA_Choreutidae_Hemerophila_felis_HFEL
|     |      |                                                              |     |                                     /------ SESIOIDEA_Castniidae_Amauta_cacica_AMCA
|     |      |                                                              |     |                              /-100--+------ SESIOIDEA_Castniidae_Synemon_plana_SPLA
|     |      \------------------------------4-------------------------------+     \-------------23---------------+------------- COSSOIDEA_Cossidae_Prionoxystus_robiniae_PROB2
|     |                                                                     |                                           /------ ZYGAENOIDEA_Epipyropidae_Fulgoraecia_exigua_ESP2
100   |                                                                     \---------------------31--------------------+------ COSSOIDEA_Cossidae_Zeuzera_coffeae_ZCOF
|     |                                                                                                                 /------ YPONOMEUTOIDEA_Yponomeutidae_Atteva_punctella_ATPU2
|     |                                                                                                          /--60--+------ YPONOMEUTOIDEA_Plutellidae_Eucalantica_sp_YSP
|     \----------------------------------------------------79----------------------------------------------------+------------- GRACILLARIOIDEA_Gracillariidae_Caloptilia_bimaculatella_CBIM
|                                                                                                                       /------ TINEOIDEA_Tineidae_Tineola_bisselliella_TBI3
\-----------------------------------------------------------------------------------------------------------------------+------ TINEOIDEA_Tineidae_Tinea_columbariella_TCO2
```
