# Supplementary material for: Toward reconstructing the evolution of advanced moths and butterflies (Lepidoptera: Ditrysia): an initial molecular study
Source: BMC Evol Biol. 2009 Dec 2;9:280. doi: 10.1186/1471-2148-9-280 (PMC2796670; doi:10.1186/1471-2148-9-280)
Supplement: Additional file 8 — Effects of compositional heterogeneity on inferred relationships, compared between nt3 and noLRall2 + nt2. Part A. Analysis of variable nt3 characters. Part B. Analysis of noLRall2 + nt2 characters. [file 1471-2148-9-280-S8.PDF]

Additional File 8 – Effects of compositional heterogeneity on inferred relationships, compared between nt3 and noLRall2 + nt2

Part A. Analysis of variable nt3 characters. Horizontal phylogram without taxon labels is NJ tree based on compositional distance (Euclidean distance calculated on proportions of the four bases, treated as independent characters). The compositional distance scale is shown to the right of the Neighbor-Joining tree. Map of missing gene regions (if any) for each taxon on the phylogram is shown directly below, in the chart below the phylogram. In top row, selected regions (bold lines) of composition-based tree are compared in detail to corresponding trees inferred from nucleotide substitutions by distance (NJ on ML distance using GTR model) and ML (GTR + G model) methods. Bootstrap values ≥ 50% shown to left of nodes.

Part B. Analysis of noLRall2 + nt2 characters. As above (with same numerical meaning for tick marks on compositional distance scale), except that no comparisons of composition-based phylogram to substitution-based trees are shown.

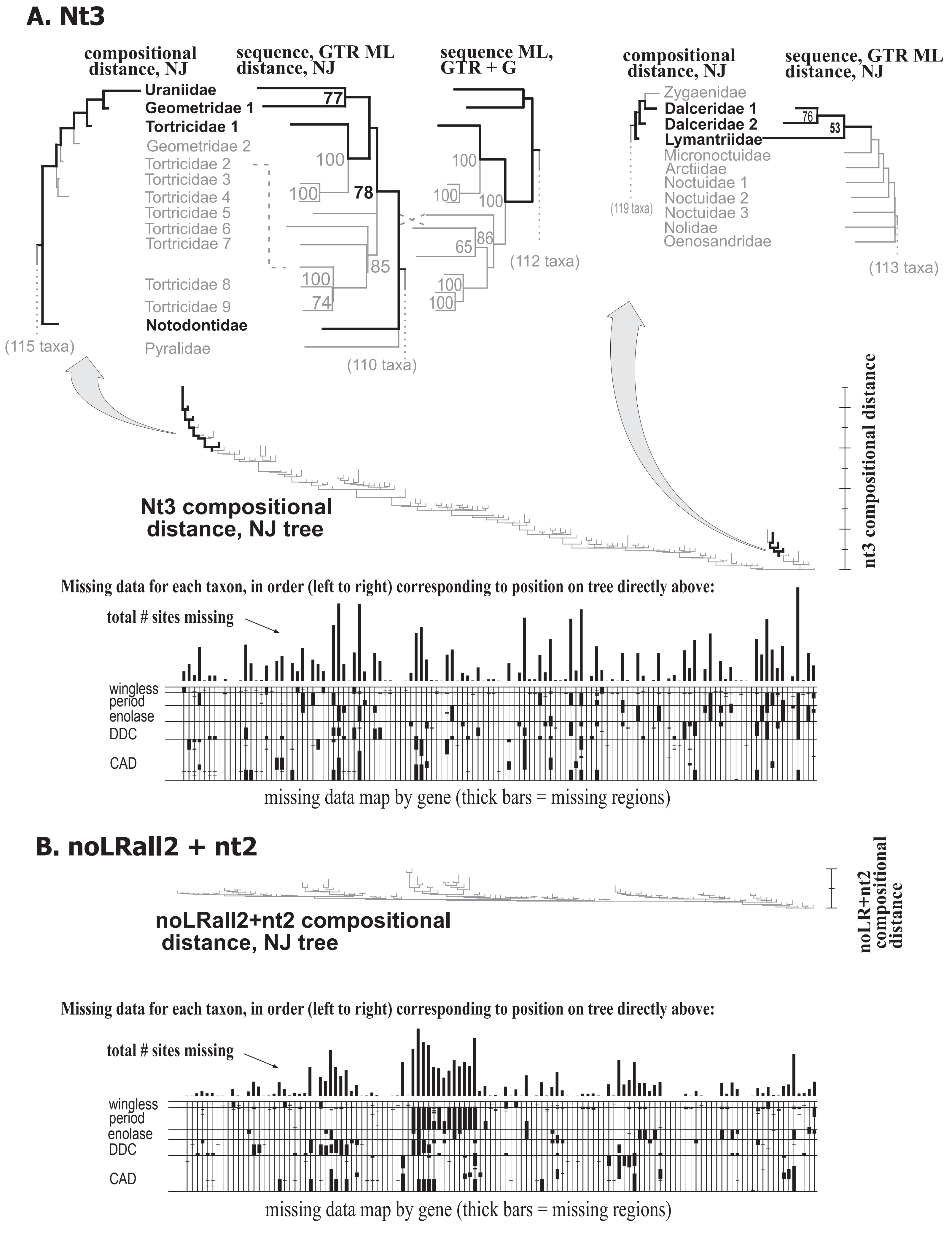
A. Nt3
compositional distance, NJ
sequence, GTR ML distance, NJ
sequence ML, GTR + G
compositional distance, NJ
sequence, GTR ML distance, NJ
Uraniidae
Geometridae 1
Tortricidae 1
Geometridae 2
Tortricidae 2
Tortricidae 3
Tortricidae 4
Tortricidae 5
Tortricidae 6
Tortricidae 7
Tortricidae 8
Tortricidae 9
Notodontidae
Pyralidae
(115 taxa)
77
100
100
78
85
100
74
(110 taxa)
100
100
100
65
86
100
100
(112 taxa)
Zygaenidae
Dalceridae 1
Dalceridae 2
Lymantriidae
Micronoctuidae
Arctiidae
Noctuidae 1
Noctuidae 2
Noctuidae 3
Nolidae
Oenosandridae
(119 taxa)
76
53
(113 taxa)
Nt3 compositional distance, NJ tree
nt3 compositional distance
Missing data for each taxon, in order (left to right) corresponding to position on tree directly above:
total # sites missing
wingless
period
enolase
DDC
CAD
missing data map by gene (thick bars = missing regions)
B. noLRall2 + nt2
noLRall2+nt2 compositional distance, NJ tree
noLR+nt2 compositional distance
Missing data for each taxon, in order (left to right) corresponding to position on tree directly above:
total # sites missing
wingless
period
enolase
DDC
CAD
missing data map by gene (thick bars = missing regions)
