# Supplementary material for: Toward reconstructing the evolution of advanced moths and butterflies (Lepidoptera: Ditrysia): an initial molecular study
Source: BMC Evol Biol. 2009 Dec 2;9:280. doi: 10.1186/1471-2148-9-280 (PMC2796670; doi:10.1186/1471-2148-9-280)
Supplement: Additional file 9 — 123-taxon ML tree & bootstrap consensus tree for nt3. Part A. nt3 best ML tree found in 10,000 replicate GARLI searches, GTR + G + I model, phylogram format. Part B. nt3, bootstrap 50% majority rule consensus tree, generated in PAUP, from 1000 GARLI ML bootstrap replicates, GTR + G + I model. [file 1471-2148-9-280-S9.PDF]

Additional File 8, Part A. Nt3 best ML tree found in 10,000 replicate GARLI searches, GTR + G + I model.

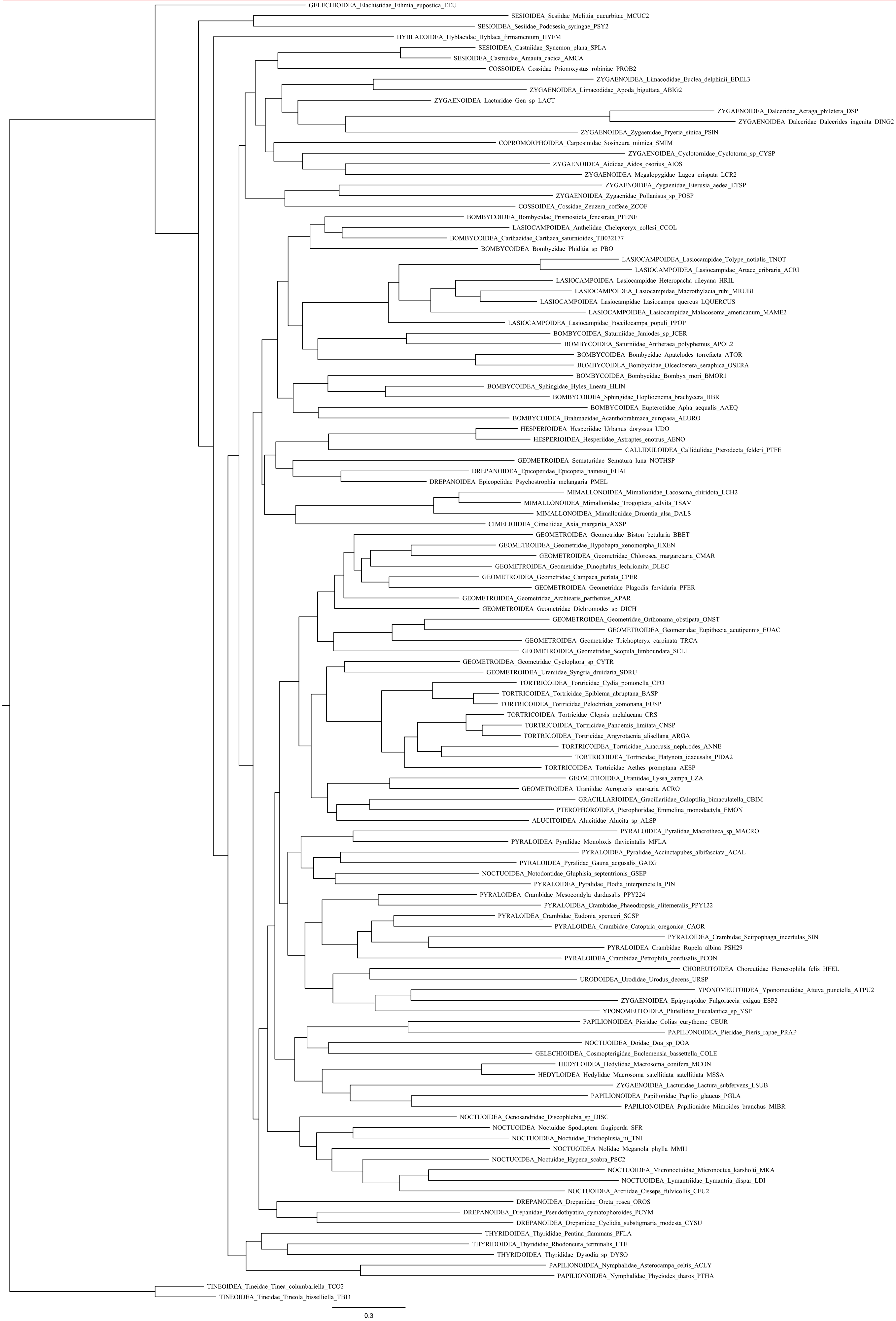

Additional File 8, Part B. Nt3, bootstrap majority rule consensus tree (LE option on), generated in PAUP, from 1000 GARLI ML bootstrap replicates, GTR +G+I model. Bootstrap values are embedded in branches.

```
/--------------------------------------------------------------------------- TINEOIDEA_Tineidae_Tineola_bisselliella_TBI3
|
|                                                                     /----- BOMBYCOIDEA_Eupterotidae_Apha_aequalis_AAEQ
|                               /-----------------12------------------+
|                               |                                     \----- BOMBYCOIDEA_Brahmaeidae_Acanthobrahmaea_europaea_AEURO
|                               |
|                               |                                     /----- LASIOCAMPOIDEA_Lasiocampidae_Artace_cribraria_ACRI
|                               |                     /------100------+
|                               |                     |               \----- LASIOCAMPOIDEA_Lasiocampidae_Tolype_notialis_TNOT
|                               |                     |
|                               |               /-51--+         /----------- LASIOCAMPOIDEA_Lasiocampidae_Heteropacha_rileyana_HRIL
|                               |               |     |         |
|                               |               |     |    /-96-+     /----- LASIOCAMPOIDEA_Lasiocampidae_Lasiocampa_quercus_LQUERCUS
|                               |               |     |    |    \-100-+
|                          /-1--+          /100-+     \-98-+          \----- LASIOCAMPOIDEA_Lasiocampidae_Macrothylacia_rubi_MRUBI
|                          |    |          |    |          |
|                          |    |          |    |          \---------------- LASIOCAMPOIDEA_Lasiocampidae_Malacosoma_americanum_MAME2
|                          |    |          |    |
|                          |    |          |    \--------------------------- LASIOCAMPOIDEA_Lasiocampidae_Poecilocampa_populi_PPOP
|                          |    |     /-14-+
|                          |    |     |    |                          /----- BOMBYCOIDEA_Saturniidae_Antheraea_polyphemus_APOL2
|                          |    |     |    |                    /-100-+
|                          |    |     |    |                    |     \----- BOMBYCOIDEA_Saturniidae_Janiodes_sp_JCER
|                          |    |     |    \---------26---------+
|                          |    |     |                         |     /----- BOMBYCOIDEA_Bombycidae_Apatelodes_torrefacta_ATOR
|                          |    \--6--+                         \-100-+
|               /----1-----+          |                               \----- BOMBYCOIDEA_Bombycidae_Olceclostera_seraphica_OSERA
|               |          |          |
|               |          |          |                               /----- LASIOCAMPOIDEA_Anthelidae_Chelepteryx_collesi_CCOL
|               |          |          |                         /-62--+
|               |          |          |                         |     \----- BOMBYCOIDEA_Carthaeidae_Carthaea_saturnioides_TB032177
|               |          |          |                    /-63-+
|               |          |          \---------44---------+    \----------- BOMBYCOIDEA_Bombycidae_Prismosticta_fenestrata_PFENE
|               |          |                               |
|               |          |                               \---------------- BOMBYCOIDEA_Bombycidae_Phiditia_sp_PBO
|               |          |
|               |          |                                    /----------- BOMBYCOIDEA_Bombycidae_Bombyx_mori_BMOR1
|               |          |                                    |
|               |          \-----------------26-----------------+     /----- BOMBYCOIDEA_Sphingidae_Hopliocnema_brachycera_HBR
|               |                                               \-99--+
|               |                                                     \----- BOMBYCOIDEA_Sphingidae_Hyles_lineata_HLIN
|               |
|               |                                                     /----- PAPILIONOIDEA_Nymphalidae_Asterocampa_celtis_ACLY
|               |                                               /-95--+
|               |                                               |     \----- PAPILIONOIDEA_Nymphalidae_Phyciodes_tharos_PTHA
|               |                                     /---19----+
|               |                                     |         |     /----- PAPILIONOIDEA_Pieridae_Colias_eurytheme_CEUR
|               |                                     |         \-89--+
|               |                                     |               \----- PAPILIONOIDEA_Pieridae_Pieris_rapae_PRAP
|               |                               /--0--+
|               |                               |     |               /----- HEDYLOIDEA_Hedylidae_Macrosoma_conifera_MCON
|               |                               |     |    /---100----+
|               |                               |     |    |          \----- HEDYLOIDEA_Hedylidae_Macrosoma_satellitiata_satellitiata_MSSA
|               |                               |     |    |
|               |                               |     \-7--+          /----- PAPILIONOIDEA_Papilionidae_Mimoides_branchus_MIBR
|               +---------------0---------------+          |    /-88--+
|               |                               |          |    |     \----- PAPILIONOIDEA_Papilionidae_Papilio_glaucus_PGLA
|               |                               |          \-22-+
|               |                               |               \----------- ZYGAENOIDEA_Lacturidae_Lactura_subfervens_LSUB
|               |                               |
|               |                               |                     /----- GELECHIOIDEA_Cosmopterigidae_Euclemensia_bassettella_COLE
|               |                               \---------12----------+
|               |                                                     \----- NOCTUOIDEA_Doidae_Doa_sp_DOA
|               |
|               |                                                     /----- HESPERIOIDEA_Hesperiidae_Astraptes_enotrus_AENO
|               |                                               /-100-+
|               |                                               |     \----- HESPERIOIDEA_Hesperiidae_Urbanus_doryssus_UDO
|               |                                          /-19-+
|               |                                          |    \----------- CALLIDULOIDEA_Callidulidae_Pterodecta_felderi_PTFE
|               +--------------------1---------------------+
|               |                                          |          /----- GEOMETROIDEA_Sematuridae_Sematura_luna_NOTHSP
|               |                                          \----14----+
|               |                                                     \----- CHOREUTOIDEA_Choreutidae_Hemerophila_felis_HFEL
|               |
|               |                               /--------------------------- GEOMETROIDEA_Geometridae_Archiearis_parthenias_APAR
|               |                               |
|               |                          /-31-+     /--------------------- GEOMETROIDEA_Geometridae_Biston_betularia_BBET
|               |                          |    |     |
|               |                          |    |     |               /----- GEOMETROIDEA_Geometridae_Campaea_perlata_CPER
```

```
|               |                          |    \-58--+    /----88----+
|               |                          |          |    |          \----- GEOMETROIDEA_Geometridae_Plagodis_fervidaria_PFER
|               |                     /-71-+          \-49-+          /----- GEOMETROIDEA_Geometridae_Chlorosea_margaretaria_CMAR
|               |                     |    |               |    /-100-+
|               |                     |    |               |    |     \----- GEOMETROIDEA_Geometridae_Hypobapta_xenomorpha_HXEN
|               |                     |    |               \-48-+
|               |                     |    |                    \----------- GEOMETROIDEA_Geometridae_Dinophalus_lechriomita_DLEC
|               |               /-27--+    |
|               |               |     |    \-------------------------------- GEOMETROIDEA_Geometridae_Dichromodes_sp_DICH
|               |               |     |
|               |               |     |                               /----- GEOMETROIDEA_Geometridae_Eupithecia_acutipennis_EUAC
|               |               |     |                         /-89--+
|               |          /-18-+     |                         |     \----- GEOMETROIDEA_Geometridae_Orthonama_obstipata_ONST
|               |          |    |     \-----------98------------+
|               |          |    |                               \----------- GEOMETROIDEA_Geometridae_Trichopteryx_carpinata_TRCA
|               |          |    |
|               |          |    \------------------------------------------- GEOMETROIDEA_Geometridae_Scopula_limboundata_SCLI
|               |          |
|               |          |                                    /----------- TORTRICOIDEA_Tortricidae_Cydia_pomonella_CPO
|               |          |                                    |
|               |          |                    /------100------+     /----- TORTRICOIDEA_Tortricidae_Epiblema_abruptana_BASP
|               |          |                    |               \-100-+
|               |          |                    |                     \----- TORTRICOIDEA_Tortricidae_Pelochrista_zomonana_EUSP
|               |          |                    |
|               |    /--1--+                    |                     /----- TORTRICOIDEA_Tortricidae_Argyrotaenia_alisellana_ARGA
|               |    |     |               /100-+               /-100-+
|               |    |     |               |    |               |     \----- TORTRICOIDEA_Tortricidae_Pandemis_limitata_CNSP
|               |    |     |               |    |          /100-+
|               |    |     |               |    |          |    \----------- TORTRICOIDEA_Tortricidae_Clepsis_melalucana_CRS
|               |    |     |               |    |     /-37-+
|               |    |     |               |    |     |    |          /----- TORTRICOIDEA_Tortricidae_Platynota_idaeusalis_PIDA2
|               |    |     |          /-17-+    \-90--+    \----64----+
|               |    |     |          |    |          |               \----- TORTRICOIDEA_Tortricidae_Anacrusis_nephrodes_ANNE
|               |    |     |          |    |          |
|               |    |     |          |    |          \--------------------- TORTRICOIDEA_Tortricidae_Aethes_promptana_AESP
|               |    |     |          |    |
|               |    |     |          |    |                          /----- GEOMETROIDEA_Uraniidae_Syngria_druidaria_SDRU
|               +-1--+     \----2-----+    \------------32------------+
|               |    |                |                               \----- GEOMETROIDEA_Geometridae_Cyclophora_sp_CYTR
|               |    |                |
|               |    |                |                               /----- GRACILLARIOIDEA_Gracillariidae_Caloptilia_bimaculatella_CBIM
|               |    |                |                         /-49--+
|               |    |                |                         |     \----- PTEROPHOROIDEA_Pterophoridae_Emmelina_monodactyla_EMON
|               |    |                \------------4------------+
|               |    |                                          |     /----- GEOMETROIDEA_Uraniidae_Acropteris_sparsaria_ACRO
|               |    |                                          \-57--+
|               |    |                                                \----- GEOMETROIDEA_Uraniidae_Lyssa_zampa_LZA
|               |    |
|               |    |                                                /----- ALUCITOIDEA_Alucitidae_Alucita_sp_ALSP
|               |    \-----------------------15-----------------------+
|          /-2--+                                                     \----- URODOIDEA_Urodidae_Urodus_decens_URSP
|          |    |
|          |    |                                          /---------------- CIMELIOIDEA_Cimeliidae_Axia_margarita_AXSP
|          |    |                                          |
|          |    |                                     /-8--+    /----------- MIMALLONOIDEA_Mimallonidae_Druentia_alsa_DALS
|          |    |                                     |    |    |
|          |    |                                     |    \100-+     /----- MIMALLONOIDEA_Mimallonidae_Lacosoma_chiridota_LCH2
|          |    +------------------0------------------+         \-63--+
|          |    |                                     |               \----- MIMALLONOIDEA_Mimallonidae_Trogoptera_salvita_TSAV
|          |    |                                     |
|          |    |                                     |               /----- DREPANOIDEA_Drepanidae_Pseudothyatira_cymatophoroides_PCYM
|          |    |                                     \------56-------+
|          |    |                                                     \----- DREPANOIDEA_Drepanidae_Cyclidia_substigmaria_modesta_CYSU
|          |    |
|          |    |                                                     /----- NOCTUOIDEA_Lymantriidae_Lymantria_dispar_LDI
|          |    |                                               /-61--+
|          |    |                                               |     \----- NOCTUOIDEA_Micronoctuidae_Micronoctua_karsholti_MKA
|          |    |                                          /-98-+
|          |    |                                     /-95-+    \----------- NOCTUOIDEA_Arctiidae_Cisseps_fulvicollis_CFU2
|          |    |                                     |    |
|          |    |                               /-63--+    \---------------- NOCTUOIDEA_Noctuidae_Hypena_scabra_PSC2
|          |    |                               |     |
|          |    |                               |     \--------------------- NOCTUOIDEA_Nolidae_Meganola_phylla_MMI1
|          |    |                          /-70-+
|          |    |                          |    |                     /----- NOCTUOIDEA_Noctuidae_Spodoptera_frugiperda_SFR
|          |    |                     /-50-+    \---------98----------+
|          |    |                     |    |                          \----- NOCTUOIDEA_Noctuidae_Trichoplusia_ni_TNI
|          |    |                     |    |
|          |    +----------7----------+    \-------------------------------- NOCTUOIDEA_Oenosandridae_Discophlebia_sp_DISC
|          |    |                     |
|          |    |                     |                               /----- DREPANOIDEA_Epicopeiidae_Epicopeia_hainesii_EHAI
|          |    |                     \--------------98---------------+
|          |    |                                                     \----- DREPANOIDEA_Epicopeiidae_Psychostrophia_melangaria_PMEL
|          |    |
```

```
|          |    |                                                     /----- DREPANOIDEA_Drepanidae_Oreta_rosea_OROS
|          |    +--------------------------8--------------------------+
|          |    |                                                     \----- COPROMORPHOIDEA_Carposinidae_Sosineura_mimica_SMIM
|          |    |
|          |    |                                                     /----- PYRALOIDEA_Pyralidae_Plodia_interpunctella_PIN
|          |    |                                               /-22--+
|          |    |                                               |     \----- NOCTUOIDEA_Notodontidae_Gluphisia_septentrionis_GSEP
|          |    |                                          /-8--+
|          |    |                                          |    |     /----- PYRALOIDEA_Pyralidae_Accinctapubes_albifasciata_ACAL
|          |    |                                          |    \-30--+
|          |    +--------------------2---------------------+          \----- PYRALOIDEA_Pyralidae_Gauna_aegusalis_GAEG
|          |    |                                          |
|          |    |                                          |          /----- PYRALOIDEA_Pyralidae_Macrotheca_sp_MACRO
|          |    |                                          \----44----+
|          |    |                                                     \----- PYRALOIDEA_Pyralidae_Monoloxis_flavicintalis_MFLA
|          |    |
|          |    |                                                     /----- PYRALOIDEA_Crambidae_Phaeodropsis_alitemeralis_PPY122
|          |    |                                     /------98-------+
|          |    |                                     |               \----- PYRALOIDEA_Crambidae_Mesocondyla_dardusalis_PPY224
|          |    |                                     |
|          |    |                                     |               /----- PYRALOIDEA_Crambidae_Rupela_albina_PSH29
|    /-18--+    |                               /-63--+         /-98--+
|    |     |    |                               |     |         |     \----- PYRALOIDEA_Crambidae_Scirpophaga_incertulas_SIN
|    |     |    |                               |     |    /-57-+
|    |     |    |                               |     |    |    |     /----- PYRALOIDEA_Crambidae_Catoptria_oregonica_CAOR
|    |     |    |                               |     |    |    \-78--+
|    |     |    |                               |     \-97-+          \----- PYRALOIDEA_Crambidae_Eudonia_spenceri_SCSP
|    |     |    +---------------1---------------+          |
|    |     |    |                               |          \---------------- PYRALOIDEA_Crambidae_Petrophila_confusalis_PCON
|    |     |    |                               |
|    |     |    |                               |                     /----- YPONOMEUTOIDEA_Yponomeutidae_Atteva_punctella_ATPU2
|    |     |    |                               |               /-26--+
|    |     |    |                               |               |     \----- ZYGAENOIDEA_Epipyropidae_Fulgoraecia_exigua_ESP2
|    |     |    |                               \------11-------+
|    |     |    |                                               \----------- YPONOMEUTOIDEA_Plutellidae_Eucalantica_sp_YSP
|    |     |    |
|    |     |    |                                                     /----- ZYGAENOIDEA_Limacodidae_Apoda_biguttata_ABIG2
|    |     |    |                                               /-98--+
|    |     |    |                                               |     \----- ZYGAENOIDEA_Limacodidae_Euclea_delphinii_EDEL3
|    |     |    |                                     /---11----+
|    |     |    |                                     |         \----------- ZYGAENOIDEA_Lacturidae_Gen_sp_LACT
|    |     |    |                                     |
|    |     |    |                               /--1--+               /----- SESIOIDEA_Castniidae_Amauta_cacica_AMCA
|    |     |    |                               |     |         /-100-+
|    |     |    |                               |     |         |     \----- SESIOIDEA_Castniidae_Synemon_plana_SPLA
|    |     |    |                               |     |    /-14-+
|    |     |    |                               |     \-2--+    \----------- ZYGAENOIDEA_Zygaenidae_Pryeria_sinica_PSIN
|    |     |    +---------------0---------------+          |
|    |     |    |                               |          \---------------- COSSOIDEA_Cossidae_Prionoxystus_robiniae_PROB2
+100-+     |    |                               |
|    |     |    |                               |               /----------- ZYGAENOIDEA_Cyclotornidae_Cyclotorna_sp_CYSP
|    |     |    |                               |               |
|    |     |    |                               \------10-------+     /----- ZYGAENOIDEA_Megalopygidae_Lagoa_crispata_LCR2
|    |     |    |                                               \-52--+
|    |     |    |                                                     \----- ZYGAENOIDEA_Aididae_Aidos_osorius_AIOS
|    |     |    |
|    |     |    |                                                     /----- ZYGAENOIDEA_Dalceridae_Dalcerides_ingenita_DING2
|    |     |    |                                               /-100-+
|    |     |    |                                               |     \----- ZYGAENOIDEA_Dalceridae_Acraga_philetera_DSP
|    |     |    |                                          /-17-+
|    |     |    |                                          |    \----------- COSSOIDEA_Cossidae_Zeuzera_coffeae_ZCOF
|    |     |    |                                          |
|    |     |    +--------------------0---------------------+          /----- ZYGAENOIDEA_Zygaenidae_Eterusia_aedea_ETSP
|    |     |    |                                          |    /-28--+
|    |     |    |                                          |    |     \----- ZYGAENOIDEA_Zygaenidae_Pollanisus_sp_POSP
|    |     |    |                                          \-2--+
|    |     |    |                                               \----------- HYBLAEOIDEA_Hyblaeidae_Hyblaea_firmamentum_HYFM
|    |     |    |
|    |     |    |                                                     /----- THYRIDOIDEA_Thyrididae_Dysodia_sp_DYSO
|    |     |    |                                               /-27--+
|    |     |    |                                               |     \----- THYRIDOIDEA_Thyrididae_Rhodoneura_terminalis_LTE
|    |     |    \-----------------------9-----------------------+
|    |     |                                                    \----------- THYRIDOIDEA_Thyrididae_Pentina_flammans_PFLA
|    |     |
|    |     |                                                          /----- SESIOIDEA_Sesiidae_Melittia_cucurbitae_MCUC2
|    |     \----------------------------38----------------------------+
|    |                                                                \----- SESIOIDEA_Sesiidae_Podosesia_syringae_PSY2
|    |
|    \---------------------------------------------------------------------- GELECHIOIDEA_Elachistidae_Ethmia_eupostica_EEU
|
\--------------------------------------------------------------------------- TINEOIDEA_Tineidae_Tinea_columbariella_TCO2
```
