# Supplementary material for: Toward reconstructing the evolution of advanced moths and butterflies (Lepidoptera: Ditrysia): an initial molecular study
Source: BMC Evol Biol. 2009 Dec 2;9:280. doi: 10.1186/1471-2148-9-280 (PMC2796670; doi:10.1186/1471-2148-9-280)
Supplement: Additional file 4 — 123-taxon ML tree & bootstrap consensus tree for nt123. Part A: nt123, best ML tree found in 10,000 replicate GARLI searches, GTR + G + I model, phylogram format. Part B: nt123, majority rule consensus tree from 1000 GARLI ML bootstrap replicates, generated in PAUP. [file 1471-2148-9-280-S4.PDF]

Additional File 4, Part A. nt123best ML tree found in 10,000 replicate GARLI searches, GTR + G + I model.

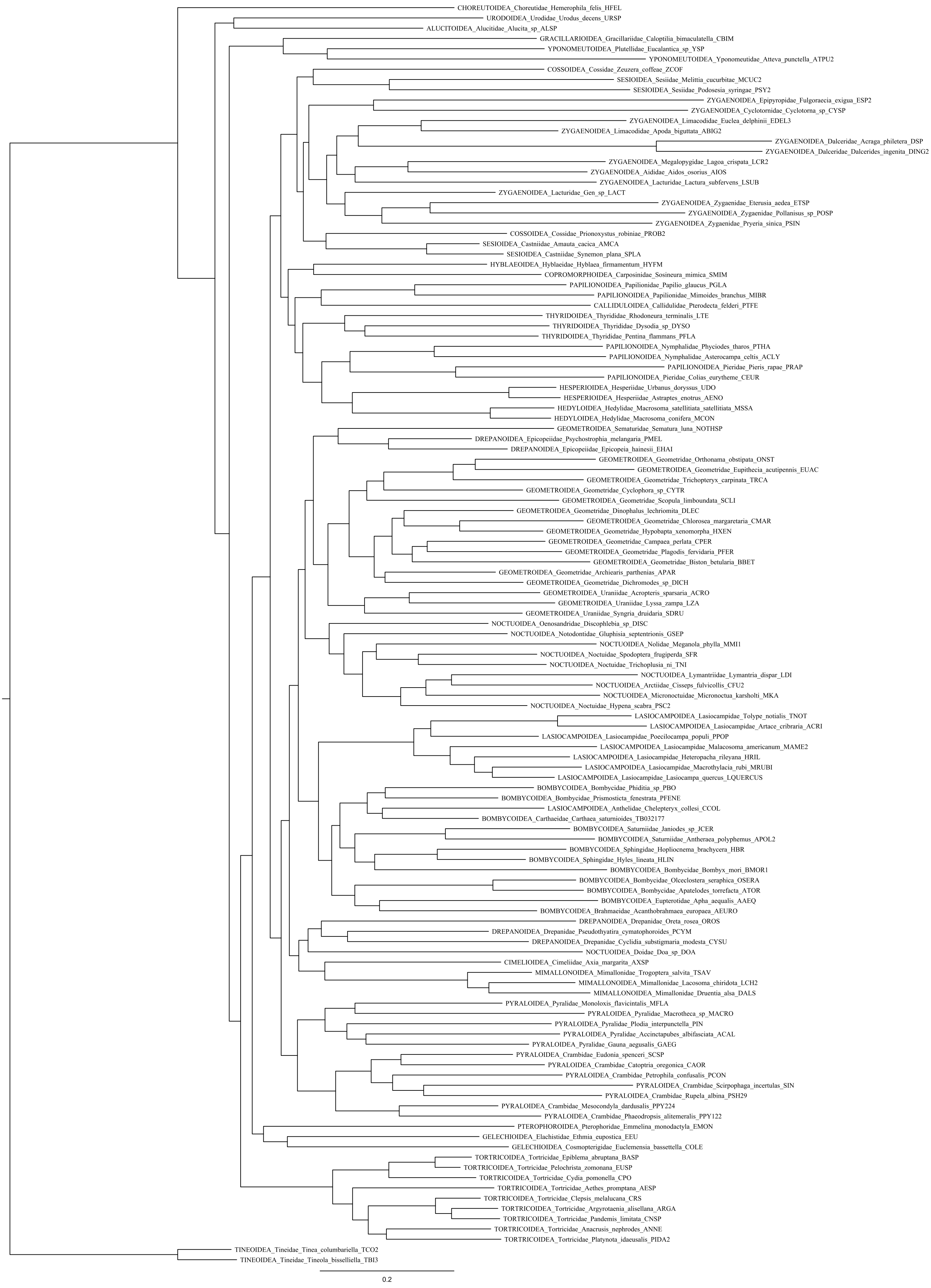

Additional File 4, Part B. nt123, bootstrap majority rule consensus tree (with LE option on), generated in PAUP, from 1000 GARLI ML bootstrap replicates, GTR +G+I model. Bootstrap values are embedded in branches.

```
                                                          /--68---+------- GEOMETROIDEA_Geometridae_Plagodis_fervidaria_PFER
                                                   /-100--+--------------- GEOMETROIDEA_Geometridae_Biston_betularia_BBET
                                                   |              /------- GEOMETROIDEA_Geometridae_Chlorosea_margaretaria_CMAR
                                            /--98--+      /--100--+------- GEOMETROIDEA_Geometridae_Hypobapta_xenomorpha_HXEN
                                            |      \--66--+--------------- GEOMETROIDEA_Geometridae_Dinophalus_lechriomita_DLEC
                                     /-100--+                     /------- GEOMETROIDEA_Geometridae_Archiearis_parthenias_APAR
                                     |      \---------54----------+------- GEOMETROIDEA_Geometridae_Dichromodes_sp_DICH
                                     |                            /------- GEOMETROIDEA_Geometridae_Eupithecia_acutipennis_EUAC
                             /--95---+                    /--53---+------- GEOMETROIDEA_Geometridae_Orthonama_obstipata_ONST
                             |       |             /-100--+--------------- GEOMETROIDEA_Geometridae_Trichopteryx_carpinata_TRCA
                             |       \-----73------+              /------- GEOMETROIDEA_Geometridae_Cyclophora_sp_CYTR
                      /--53--+                     \------62------+------- GEOMETROIDEA_Geometridae_Scopula_limboundata_SCLI
                      |      |                                    /------- GEOMETROIDEA_Uraniidae_Acropteris_sparsaria_ACRO
                      |      |                            /--94---+------- GEOMETROIDEA_Uraniidae_Lyssa_zampa_LZA
               /--16--+      \------------83--------------+--------------- GEOMETROIDEA_Uraniidae_Syngria_druidaria_SDRU
               |      |                                           /------- DREPANOIDEA_Epicopeiidae_Epicopeia_hainesii_EHAI
               |      |                                   /--100--+------- DREPANOIDEA_Epicopeiidae_Psychostrophia_melangaria_PMEL
               |      \----------------67-----------------+--------------- GEOMETROIDEA_Sematuridae_Sematura_luna_NOTHSP
               |                                                  /------- NOCTUOIDEA_Lymantriidae_Lymantria_dispar_LDI
               |                                          /--89---+------- NOCTUOIDEA_Arctiidae_Cisseps_fulvicollis_CFU2
       /---8---+                                   /--98--+--------------- NOCTUOIDEA_Micronoctuidae_Micronoctua_karsholti_MKA
       |       |                            /-100--+---------------------- NOCTUOIDEA_Noctuidae_Hypena_scabra_PSC2
       |       |                            |                     /------- NOCTUOIDEA_Noctuidae_Spodoptera_frugiperda_SFR
       |       |                     /--94--+             /--100--+------- NOCTUOIDEA_Noctuidae_Trichoplusia_ni_TNI
       |       |             /--58---+      \-----59------+--------------- NOCTUOIDEA_Nolidae_Meganola_phylla_MMI1
       |       \-----83------+       \------------------------------------ NOCTUOIDEA_Notodontidae_Gluphisia_septentrionis_GSEP
       |                     \-------------------------------------------- NOCTUOIDEA_Oenosandridae_Discophlebia_sp_DISC
       |                                                          /------- BOMBYCOIDEA_Sphingidae_Hopliocnema_brachycera_HBR
       |                                                  /--100--+------- BOMBYCOIDEA_Sphingidae_Hyles_lineata_HLIN
       |                                           /--40--+--------------- BOMBYCOIDEA_Bombycidae_Bombyx_mori_BMOR1
       |                                    /--33--+              /------- BOMBYCOIDEA_Saturniidae_Antheraea_polyphemus_APOL2
       |                                    |      \-----100------+------- BOMBYCOIDEA_Saturniidae_Janiodes_sp_JCER
/--2---+                             /--30--+                     /------- LASIOCAMPOIDEA_Anthelidae_Chelepteryx_collesi_CCOL
|      |                             |      |             /--61---+------- BOMBYCOIDEA_Carthaeidae_Carthaea_saturnioides_TB032177
|      |                             |      \-----88------+       /------- BOMBYCOIDEA_Bombycidae_Phiditia_sp_PBO
|      |                     /--35---+                    \--52---+------- BOMBYCOIDEA_Bombycidae_Prismosticta_fenestrata_PFENE
|      |                     |       |                            /------- BOMBYCOIDEA_Eupterotidae_Apha_aequalis_AAEQ
|      |                     |       |                    /--86---+------- BOMBYCOIDEA_Brahmaeidae_Acanthobrahmaea_europaea_AEURO
|      |                     |       \---------73---------+       /------- BOMBYCOIDEA_Bombycidae_Apatelodes_torrefacta_ATOR
|      |                     |                            \--100--+------- BOMBYCOIDEA_Bombycidae_Olceclostera_seraphica_OSERA
|      |              /--58--+                                    /------- LASIOCAMPOIDEA_Lasiocampidae_Lasiocampa_quercus_LQUERCUS
```

```
                             |      |              |      |                            /--50---+------- LASIOCAMPOIDEA_Lasiocampidae_Macrothylacia_rubi_MRUBI
                             |      |              |      |                     /-100--+--------------- LASIOCAMPOIDEA_Lasiocampidae_Heteropacha_rileyana_HRIL
                      /--9---+      |              |      |              /-100--+---------------------- LASIOCAMPOIDEA_Lasiocampidae_Malacosoma_americanum_MAME2
                      |      |      \------23------+      |              |                     /------- LASIOCAMPOIDEA_Lasiocampidae_Artace_cribraria_ACRI
                      |      |                     |      \-----100------+             /--100--+------- LASIOCAMPOIDEA_Lasiocampidae_Tolype_notialis_TNOT
                      |      |                     |                     \-----58------+--------------- LASIOCAMPOIDEA_Lasiocampidae_Poecilocampa_populi_PPOP
                      |      |                     \--------------------------------------------------- CIMELIOIDEA_Cimeliidae_Axia_margarita_AXSP
                      |      |                                                                 /------- MIMALLONOIDEA_Mimallonidae_Lacosoma_chiridota_LCH2
                      |      |                                                         /--53---+------- MIMALLONOIDEA_Mimallonidae_Trogoptera_salvita_TSAV
                      |      |                                                  /-100--+--------------- MIMALLONOIDEA_Mimallonidae_Druentia_alsa_DALS
                      |      |                                           /--21--+---------------------- NOCTUOIDEA_Doidae_Doa_sp_DOA
                      |      |                                           |                     /------- DREPANOIDEA_Drepanidae_Pseudothyatira_cymatophoroides_PCYM
              /--14---+      \---------------------3---------------------+             /--65---+------- DREPANOIDEA_Drepanidae_Cyclidia_substigmaria_modesta_CYSU
              |       |                                                  \-----37------+--------------- DREPANOIDEA_Drepanidae_Oreta_rosea_OROS
              |       |                                                                        /------- PYRALOIDEA_Crambidae_Rupela_albina_PSH29
              |       |                                                                /--80---+------- PYRALOIDEA_Crambidae_Scirpophaga_incertulas_SIN
              |       |                                                         /--80--+--------------- PYRALOIDEA_Crambidae_Petrophila_confusalis_PCON
              |       |                                                  /-100--+              /------- PYRALOIDEA_Crambidae_Catoptria_oregonica_CAOR
              |       |                                                  |      \-----100------+------- PYRALOIDEA_Crambidae_Eudonia_spenceri_SCSP
              |       |                                           /-100--+                     /------- PYRALOIDEA_Crambidae_Phaeodropsis_alitemeralis_PPY122
              |       |                                           |      \---------100---------+------- PYRALOIDEA_Crambidae_Mesocondyla_dardusalis_PPY224
              |       |                                           |                            /------- PYRALOIDEA_Pyralidae_Accinctapubes_albifasciata_ACAL
              |       \--------------------65---------------------+                    /--86---+------- PYRALOIDEA_Pyralidae_Gauna_aegusalis_GAEG
              |                                                   |             /--88--+--------------- PYRALOIDEA_Pyralidae_Plodia_interpunctella_PIN
              |                                                   \-----99------+              /------- PYRALOIDEA_Pyralidae_Macrotheca_sp_MACRO
              |                                                                 \------91------+------- PYRALOIDEA_Pyralidae_Monoloxis_flavicintalis_MFLA
       /--1---+                                                                                /------- PAPILIONOIDEA_Nymphalidae_Asterocampa_celtis_ACLY
       |      |                                                                        /--100--+------- PAPILIONOIDEA_Nymphalidae_Phyciodes_tharos_PTHA
       |      |                                                                 /--68--+       /------- PAPILIONOIDEA_Pieridae_Colias_eurytheme_CEUR
       |      |                                                                 |      \--100--+------- PAPILIONOIDEA_Pieridae_Pieris_rapae_PRAP
       |      |                                                          /--50--+              /------- HESPERIOIDEA_Hesperiidae_Astraptes_enotrus_AENO
       |      |                                                          |      |      /--100--+------- HESPERIOIDEA_Hesperiidae_Urbanus_doryssus_UDO
       |      |                                                          |      \--68--+       /------- HEDYLOIDEA_Hedylidae_Macrosoma_conifera_MCON
       |      |                                                   /--11--+             \--100--+------- HEDYLOIDEA_Hedylidae_Macrosoma_satellitiata_satellitiata_MSSA
       |      |                                                   |      |                     /------- THYRIDOIDEA_Thyrididae_Dysodia_sp_DYSO
/--2---+      |                                                   |      |             /--46---+------- THYRIDOIDEA_Thyrididae_Pentina_flammans_PFLA
|      |      |                                           /--10---+      \-----99------+--------------- THYRIDOIDEA_Thyrididae_Rhodoneura_terminalis_LTE
|      |      |                                           |       |                            /------- PAPILIONOIDEA_Papilionidae_Mimoides_branchus_MIBR
|      |      |                                           |       |                    /--100--+------- PAPILIONOIDEA_Papilionidae_Papilio_glaucus_PGLA
|      |      \---------------------5---------------------+       \---------25---------+--------------- CALLIDULOIDEA_Callidulidae_Pterodecta_felderi_PTFE
|      |                                                  |                                    /------- COPROMORPHOIDEA_Carposinidae_Sosineura_mimica_SMIM
|      |                                                  \-----------------25-----------------+------- HYBLAEOIDEA_Hyblaeidae_Hyblaea_firmamentum_HYFM
```

```
                      |      |                                                                                       /------- GELECHIOIDEA_Cosmopterigidae_Euclemensia_bassettella_COLE
                      |      |                                                                               /--32---+------- GELECHIOIDEA_Elachistidae_Ethmia_eupostica_EEU
                      |      \---------------------------------------7---------------------------------------+--------------- PTEROPHOROIDEA_Pterophoridae_Emmelina_monodactyla_EMON
                      |                                                                                              /------- TORTRICOIDEA_Tortricidae_Argyrotaenia_alisellana_ARGA
               /--4---+                                                                                      /--100--+------- TORTRICOIDEA_Tortricidae_Pandemis_limitata_CNSP
               |      |                                                                               /-100--+--------------- TORTRICOIDEA_Tortricidae_Clepsis_melalucana_CRS
               |      |                                                                        /--81--+              /------- TORTRICOIDEA_Tortricidae_Platynota_idaeusalis_PIDA2
               |      |                                                                 /--99--+      \------70------+------- TORTRICOIDEA_Tortricidae_Anacrusis_nephrodes_ANNE
               |      |                                                                 |      \----------------------------- TORTRICOIDEA_Tortricidae_Aethes_promptana_AESP
               |      |                                                         /--100--+                            /------- TORTRICOIDEA_Tortricidae_Epiblema_abruptana_BASP
               |      |                                                         |       |                    /--100--+------- TORTRICOIDEA_Tortricidae_Pelochrista_zomonana_EUSP
               |      |                                                         |       \--------100---------+--------------- TORTRICOIDEA_Tortricidae_Cydia_pomonella_CPO
               |      |                                                  /--8---+                                    /------- YPONOMEUTOIDEA_Yponomeutidae_Atteva_punctella_ATPU2
               |      |                                                  |      |                            /--78---+------- YPONOMEUTOIDEA_Plutellidae_Eucalantica_sp_YSP
               |      \------------------------2-------------------------+      \-------------50-------------+--------------- GRACILLARIOIDEA_Gracillariidae_Caloptilia_bimaculatella_CBIM
               |                                                         |                                           /------- ALUCITOIDEA_Alucitidae_Alucita_sp_ALSP
               |                                                         \--------------------16---------------------+------- URODOIDEA_Urodidae_Urodus_decens_URSP
               |                                                                                                     /------- ZYGAENOIDEA_Limacodidae_Apoda_biguttata_ABIG2
               |                                                                                             /--100--+------- ZYGAENOIDEA_Limacodidae_Euclea_delphinii_EDEL3
       /--19---+                                                                                      /--34--+       /------- ZYGAENOIDEA_Dalceridae_Dalcerides_ingenita_DING2
       |       |                                                                                      |      \--100--+------- ZYGAENOIDEA_Dalceridae_Acraga_philetera_DSP
       |       |                                                                               /--20--+              /------- ZYGAENOIDEA_Megalopygidae_Lagoa_crispata_LCR2
       |       |                                                                               |      |      /--100--+------- ZYGAENOIDEA_Aididae_Aidos_osorius_AIOS
       |       |                                                                               |      \--22--+--------------- ZYGAENOIDEA_Lacturidae_Lactura_subfervens_LSUB
       |       |                                                                        /--76--+                     /------- ZYGAENOIDEA_Zygaenidae_Eterusia_aedea_ETSP
       |       |                                                                        |      |             /--65---+------- ZYGAENOIDEA_Zygaenidae_Pollanisus_sp_POSP
       |       |                                                                        |      |      /--71--+--------------- ZYGAENOIDEA_Zygaenidae_Pryeria_sinica_PSIN
       |       |                                                                /--27---+      \--32--+---------------------- ZYGAENOIDEA_Lacturidae_Gen_sp_LACT
/------+       |                                                                |       |                            /------- SESIOIDEA_Castniidae_Amauta_cacica_AMCA
|      |       |                                                                |       |                    /--100--+------- SESIOIDEA_Castniidae_Synemon_plana_SPLA
|      |       |                                                         /--13--+       \---------60---------+--------------- COSSOIDEA_Cossidae_Prionoxystus_robiniae_PROB2
|      |       |                                                         |      |                                    /------- SESIOIDEA_Sesiidae_Melittia_cucurbitae_MCUC2
|      |       |                                                         |      |                            /--100--+------- SESIOIDEA_Sesiidae_Podosesia_syringae_PSY2
100    |       \----------------------------8----------------------------+      \-------------26-------------+--------------- COSSOIDEA_Cossidae_Zeuzera_coffeae_ZCOF
|      |                                                                 |                                           /------- ZYGAENOIDEA_Cyclotornidae_Cyclotorna_sp_CYSP
|      |                                                                 \--------------------35---------------------+------- ZYGAENOIDEA_Epipyropidae_Fulgoraecia_exigua_ESP2
|      \--------------------------------------------------------------------------------------------------------------------- CHOREUTOIDEA_Choreutidae_Hemerophila_felis_HFEL
|                                                                                                                    /------- TINEOIDEA_Tineidae_Tineola_bisselliella_TBI3
\--------------------------------------------------------------------------------------------------------------------+------- TINEOIDEA_Tineidae_Tinea_columbariella_TCO2

paup> execute O9.GARLI
Data matrix has 123 taxa, 6633 characters
paup> set torder=left tcompress=yes maxtrees=1000 root=outgroup outroot=monophyl
paup> outgroup TINEITbi3 TINEITco2
paup> gettrees file=O9.boot.tre
```

```
1000 trees read from file
paup> contree all/strict=no majrule=yes grpfreq=no le=yes
50% Majority-rule consensus of 1000 trees
```
